# Restriction–Modification Systems in Lactic Acid Bacteria: Diversity, Mechanisms, Evolution, and Biotechnological Applications

**DOI:** 10.3390/ijms272110007

**Published:** 2026-11-08

**Authors:** Kseniya D. Bondarenko, Alina I. Selimzianova, Oksana V. Berezina, Aleksei S. Rozanov, Leonid A. Shaposhnikov

**Affiliations:** 1 Research Center for Industrial and Agricultural Biotechnologies, Sirius University of Science and Technology, 354340 Sochi, Russia; 2 National Research Center “Kurchatov Institute”, 123182 Moscow, Russia; 3 Centre of Advanced Studies, 123592 Moscow, Russia

**Keywords:** bacteriophage resistance, genetic engineering, lactic acid bacteria, *Lactiplantibacillus plantarum*, *Lactococcus lactis*, methylome, methyltransferase, phylogenomics, restriction–modification, *Streptococcus thermophilus*

## Abstract

Lactic acid bacteria (LAB) are a physiologically defined and taxonomically diverse group of Gram-positive fermentative organisms central to food production, probiotic development, and microbial biotechnology. Their continual exposure to bacteriophages and mobile DNA has selected multiple defense strategies, but the same defenses can make industrial and non-model isolates difficult to transform or edit genetically. Restriction–modification (R-M) systems are central to this conflict because they discriminate incoming DNA according to sequence and modification state, thereby influencing phage infection, plasmid transfer, transformation, and recombination. This review examines classical Type I–IV R-M systems in LAB, emphasizing systems for which specificity, genetic organization, biochemical activity, methylation, or a restriction phenotype has been experimentally determined. Other DNA-modification-associated defense systems, including bacteriophage exclusion (BREX) and DISARM, are discussed separately from classical R-M. To complement the literature synthesis, we performed an original comparative genomic analysis of 196 quality-filtered RefSeq genomes from ten diversity-oriented, approximately genus-balanced LAB groups. A 100-marker host phylogeny was integrated with independently curated PADLOC and DefenseFinder predictions. Complete predicted R-M systems were detected in 86/196 genomes for Type I, 112/195 evaluable genomes for Type II (including IIG), 30/196 for Type III, and 100/195 for Type IV. The distribution of these predictions varied markedly among the sampled LAB groups, particularly for Type IV, although the comparative panel was not designed to establish population prevalence or the evolutionary causes of these differences. Direct mechanistic evidence remains concentrated in *Lactococcus lactis* and *Streptococcus thermophilus*, underscoring the gap between genomic prediction and experimentally demonstrated function. Engineering strategies are considered in relation to the barrier they actually address: inhibitory donor methylation, absence of recipient-like protection, high recognition-site burden, inefficient DNA delivery, or recipient restriction activity. Throughout, experimentally characterized systems are distinguished from methylome-supported assignments and sequence-based predictions.

## 1. Introduction

Lactic acid bacteria are united by physiology and technological use rather than by descent from a single lineage. Food-associated LAB include *Lactococcus*, *Streptococcus*, *Leuconostoc*, *Pediococcus*, *Oenococcus*, *Weissella*, *Enterococcus*, and several genera reclassified from the former genus *Lactobacillus* in 2020, including *Lactiplantibacillus*, *Lacticaseibacillus*, and *Limosilactobacillus* [1,2]. LAB support dairy, vegetable, cereal, meat, wine, and silage fermentations and are increasingly developed as microbial cell factories and delivery platforms [3,4]. The LAB designation is not a blanket safety category, though: enterococci participate in food fermentations but also include opportunistic pathogens and strains carrying clinically relevant antimicrobial-resistance and virulence determinants, so safety is assessed at species and strain level [5].

Bacteriophages and other mobile genetic elements exert persistent pressure on LAB populations. In dairy production, infection of a starter culture can delay acidification, interrupt fermentation, and reduce product quality. LAB therefore employ layered defenses spanning the infection cycle, including receptor variation, superinfection exclusion, restriction-modification, CRISPR-Cas, abortive infection, and toxin-antitoxin-associated systems. Additional defense-island modules include bacteriophage exclusion (BREX), the defense island system associated with restriction-modification (DISARM), and cyclic-oligonucleotide-based antiphage signaling systems (CBASS) [6,7,8]. R-M systems are particularly relevant to genome engineering because they act on foreign DNA after its entry into the cell.

Classical Types I–III combine sequence recognition with host-protective methylation and restriction of inadequately modified DNA, whereas Type IV restriction enzymes preferentially attack modified substrates and lack a cognate protective methyltransferase [9,10,11]. Consequently, the fate of a phage genome, plasmid, recombineering substrate, or genome-editing vector depends not only on its sequence but also on the modification state inherited from its previous host.

This creates an important engineering trade-off. R-M systems can protect natural and industrial cultures from phages and constrain unwanted mobile DNA, yet the same systems can destroy plasmids, editing vectors, and synthetic constructs introduced experimentally. In LAB isolates that are difficult to transform, restriction can reduce transformation efficiency by orders of magnitude or prevent recovery altogether; however, restriction is only one of several possible bottlenecks. Cell-envelope permeability, DNA delivery, plasmid replication, selectable-marker expression, and construct toxicity must be considered separately [4,12,13,14].

Evidence for R-M distribution and activity remains fragmentary across LAB. Several classical lactococcal and streptococcal systems have been resolved genetically or biochemically, whereas much of the apparent diversity in other food-associated taxa is supported only by methylome data or genome annotation. We therefore distinguish four evidence levels throughout this review: direct biochemical or genetic characterization, experimentally measured restriction/modification phenotypes, methylome-supported activity, and sequence-based prediction. This distinction is essential when moving from catalogs of candidate genes to claims about phage resistance, DNA-transfer barriers, or industrial function.

## 2. Historical and Conceptual Background

Host-controlled restriction and modification were first recognized through bacteriophage experiments: phage propagated in one bacterial host could be restricted in another, whereas rare progeny that survived acquired a host-dependent modification and subsequently infected that host more efficiently. These observations demonstrated that infectivity could change without a stable mutation in the phage and led to the discovery of paired restriction and DNA-modification activities [15,16,17].

LAB research connected these mechanisms directly to dairy phage biology. Studies in *Lactococcus lactis* identified plasmid-dependent restriction and modification phenotypes and showed that resident plasmids can influence both phage plating efficiency and survival of transforming DNA. LlaAI and LlaBI became early Type II examples, and LlaFI provided the first well-characterized Type III R-M system from a Gram-positive bacterium [18,19,20]. These studies also established a theme that remains important in industrial strains: R-M determinants frequently reside on accessory replicons rather than solely on the chromosome.

R-M systems have four overlapping biological roles [9,10,21,22]:

(1) Defense against phage genomes and other incoming DNA;
(2) Epigenetic marking of host DNA through sequence-specific methylation;
(3) Control of gene flow by restricting transformation, conjugation, plasmid transfer, or recombination between methylation-incompatible lineages;
(4) Selfish or addiction-like maintenance, because loss of protective methylation while restriction activity persists can damage the chromosome and reduce survival.

In LAB, these roles intersect with phage-rich production environments, plasmid-rich genomes, and deliberate genetic manipulation. R-M systems are therefore best viewed as variable components of an ecological and industrial defense repertoire rather than fixed housekeeping functions.

## 3. Classification and Molecular Mechanisms of R-M Systems

R-M systems are conventionally divided into Types I–IV according to subunit composition, recognition-site architecture, cofactors, methylation pattern, and cleavage behavior [11,23,24]. Classification from sequence architecture is useful but does not establish expression, motif specificity, restriction activity, or phage phenotype. A complete *hsdRMS* cassette, for example, supports a Type I prediction without demonstrating that the locus is active in its native strain.

### 3.1. Type I R-M Systems

Type I systems are multisubunit molecular machines built from HsdR, HsdM, and HsdS. Two target-recognition domains in HsdS specify a bipartite asymmetric motif separated by a spacer; HsdM provides methyltransferase activity, and HsdR supplies ATPase, translocase, and nuclease functions. HsdM-HsdS complexes can methylate DNA without HsdR, whereas restriction requires the assembled restriction complex. Cleavage is not fixed immediately beside the motif: ATP-dependent translocation precedes breakage, often at variable positions distant from the recognition site [11,25]. This behavior makes Type I systems difficult to confirm by a conventional diagnostic digest. Genome organization and methylome data can identify plausible loci, but functional assignment is strengthened by gene inactivation, complementation, motif assignment, or direct measurement of restriction. In *L. lactis*, HsdS target-recognition domains have been experimentally exchanged to generate new sequence specificities without replacing the core restriction and methylation machinery, illustrating a modular route to rapid specificity evolution [26].

Mechanistically, Type I systems are also a useful reminder that locus presence and barrier specificity are not equivalent. HsdM uses S-adenosylmethionine as the methyl-group donor, whereas restriction couples HsdR ATPase activity to DNA translocation and divalent-metal-dependent nuclease chemistry [25]. Because cleavage follows translocation and stalling rather than a fixed offset from the recognition site, the same recognition motif can generate breaks at variable positions. The modular HsdS subunit adds another layer of variability: strains with similar *hsdRMS* backbones can impose different sequence barriers when their specificity domains differ, and specificity can change without gain or loss of the complete locus [25,26]. For comparative genomics, a Type I call therefore identifies a mechanistic class but not the recognition repertoire actually experienced by an incoming plasmid or phage.

### 3.2. Type II R-M Systems

Type II systems are the most structurally diverse R-M class and include most restriction enzymes used in routine molecular biology. In the classical arrangement, separate restriction endonucleases (REases) and DNA methyltransferases (MTases) recognize the same short motif, and cleavage occurs at or near that sequence. Type IIP enzymes typically recognize palindromic sites, Type IIS enzymes cleave at a defined distance from an asymmetric site, and Type IIG proteins combine restriction and methylation functions in a single polypeptide. These mechanistic subtypes cannot be assigned reliably from the number or orientation of open reading frames alone [23,27].

The breadth of Type II is biologically important—in classical R-M pairs, the MTase uses S-adenosylmethionine to protect the cognate site, while the corresponding REase commonly uses a divalent metal ion such as Mg^2+^ for phosphodiester-bond cleavage [23,27]. Yet the spatial logic differs sharply among subtypes: a Type IIP system can make recognition-site number and exact site sequence directly relevant to construct design, whereas Type IIS and Type IIG systems introduce asymmetric recognition, offset cleavage, or fused restriction-methylation activities. Consequently, the annotation “Type II” alone says little about the practical barrier posed by a strain—motif identity, methylated base, cleavage geometry, and whether restriction and modification reside in separate or fused proteins all determine how the system will affect phages or engineered DNA [23,27].

Several lactococcal Type II systems have experimentally defined specificity and phage phenotypes. LlaAI and LlaBI from *L. lactis* subsp. *cremoris* recognize 5′-GATC-3′ and 5′-CTRYAG-3′, respectively, and cloning of LlaBI linked the locus to bacteriophage resistance [18,28]. LlaDCHI is related to the pneumococcal DpnII family, whereas plasmid-encoded LlaDII recognizes 5′-GCNGC-3′ and restricts representatives of the major lactococcal phage groups [29,30]. LlaJI, encoded by pNP40, comprises two predicted m5C MTases and two REases; its phage-resistance phenotype is strongly temperature-dependent and is most effective at 19 °C [31]. *Streptococcus thermophilus* also contains experimentally characterized Type II systems. Sth455I was isolated as a restriction endonuclease, whereas Sth368I was mapped to the integrative element ICESt1 [32,33]. These examples show that R-M activity can be carried on both plasmids and mobile chromosomal elements, but the presence of an annotated MTase alone does not demonstrate a functional R-M pair.

Taken together, the lactococcal and *S. thermophilus* examples illustrate why Type II systems resist a single ecological or engineering generalization. Some are carried on plasmids or integrative elements and can therefore enter or leave a strain with accessory DNA, while others show strong condition dependence, as in the temperature-sensitive LlaJI phenotype [18,28,29,30,31,32,33]. Thus even a biochemically valid Type II locus need not impose the same barrier under every growth condition, and two strains with comparable Type II gene counts may differ substantially in the motifs, mobile context, and effective restriction pressure encountered by incoming DNA.

### 3.3. Type III R-M Systems

Type III systems contain Mod and Res proteins. Mod recognizes and methylates an asymmetric sequence and can usually act independently, whereas restriction requires a Mod-Res complex. Methylation is commonly confined to one DNA strand, and efficient cleavage generally requires appropriately oriented recognition sites and occurs at a defined distance from the motif [34]. The requirement for communication between appropriately oriented sites gives Type III systems a geometrical constraint that is easy to miss in genome annotation, and recognition-site abundance alone is not necessarily a good predictor of restriction: orientation, spacing, and the probability that two active complexes can communicate also matter [34]. This makes Type III barriers conceptually different from a simple “site present/site absent” model and helps explain why motif maps are most informative when combined with a defined Mod-Res architecture and an experimental restriction phenotype.

LlaFI from *L. lactis* remains the best-resolved Type III system in LAB. It is encoded on plasmid pND801 by adjacent *mod* and *res* genes; the Mod protein contains methyltransferase and S-adenosylmethionine-binding motifs, whereas Res contains conserved helicase-like motifs. Endonucleolytic activity requires ATP and Mg^2+^, while S-adenosylmethionine stimulates but is not strictly required for cleavage [20,34]. Because Mod can remain functional after restriction activity has been lost, methylome evidence alone cannot establish that a predicted Type III locus still restricts incoming DNA.

### 3.4. Type IV Restriction Systems

**Figure 1:**
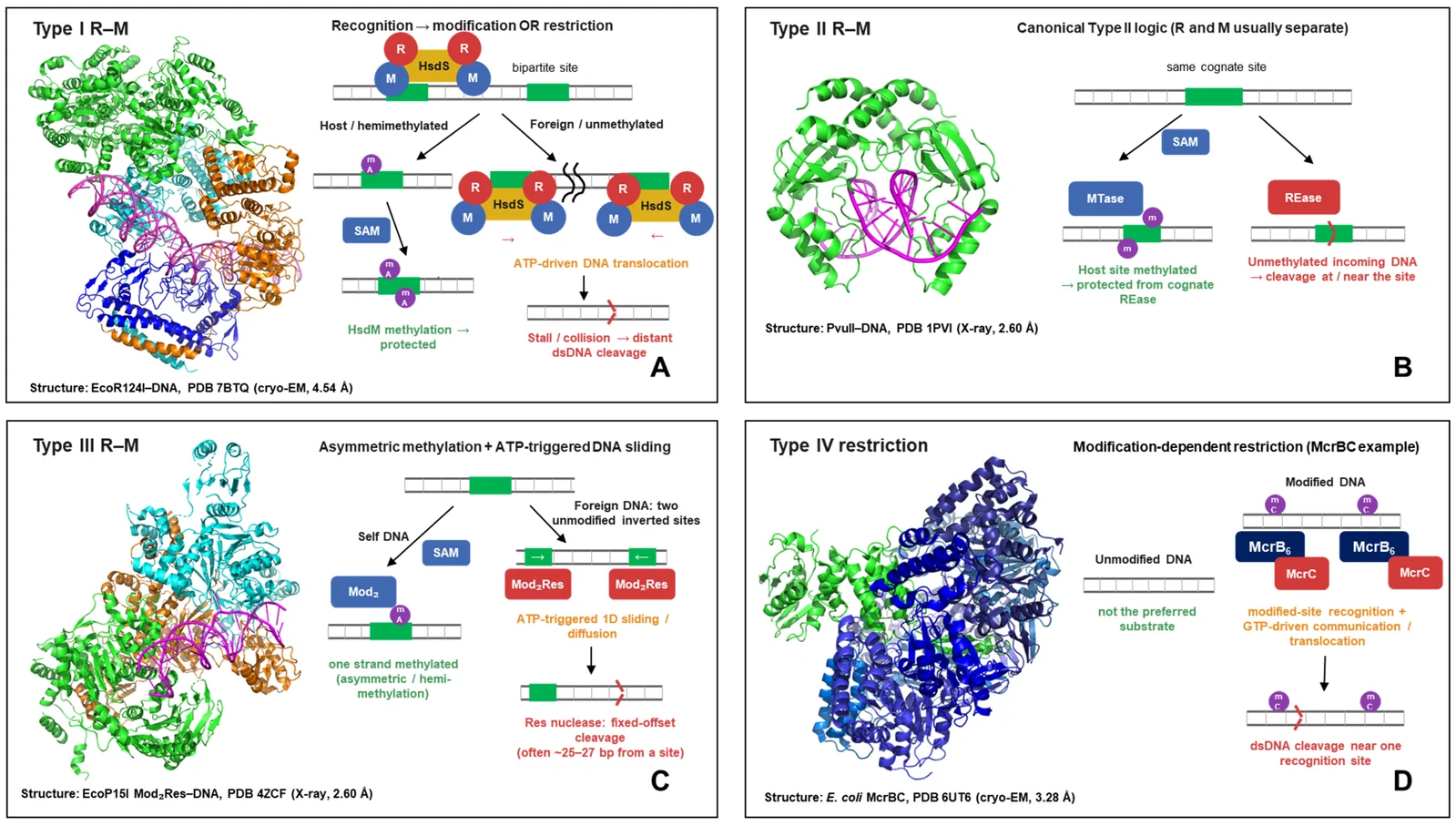
Canonical mechanisms and representative structures of Type I–IV restriction-modification systems. (**A**) Type I R-M system, (**B**) Type II R-M system, (**C**) Type III R-M system, and (**D**) Type IV R-M system. Protein structures are represented as molecular cartoons using distinct colors to distinguish visible protein chains or structural components, with magenta cartoon representing dsDNA in panels (**A**–**C**). In the mechanistic diagrams, green DNA segments indicate recognition sites and purple symbols indicate DNA methylation. Restriction, methylation, and specificity components are distinguished by their labels and schematic colors. Protein-structure colors are illustrative and should not be interpreted as an activity scale or compared quantitatively between panels.

Type IV restriction systems differ fundamentally from Types I–III because they preferentially attack modified DNA and do not require a cognate MTase for self-protection. Their substrates can include methylated adenine, methylated or hydroxymethylated cytosine, glucosylated bases, and other phage DNA modifications [10]. For genetic engineering, this reverses the usual assumption that methylated donor DNA is necessarily safer: donor methylation can instead create the recognition signal for restriction. Type IV should likewise be treated as an umbrella category rather than a single substrate rule—different Type IV families recognize different modified bases and sequence contexts, so a genomic Type IV prediction does not by itself specify which donor methylation state will be restrictive [10]. McrBC is presented in Figure 1 as one experimentally characterized example of Type-IV restriction, not as a universal mechanism for this heterogeneous class. It illustrates one well-defined solution: McrB recognizes modified cytosine-containing sites and uses GTP-dependent motor activity, while McrC provides the nuclease function; cleavage additionally depends on divalent metal ions [10]. The mechanistic diversity within the class is precisely why improved transformation with unmethylated DNA can implicate modification-dependent restriction without identifying a particular Type IV enzyme.

In LAB, one of the clearest modification-dependent transformation phenotypes has been reported in *Lactiplantibacillus plantarum*. Plasmid DNA prepared in methylation-deficient *Escherichia coli* donors produced substantially more transformants than conventionally methylated DNA in selected strains [12]. The experiment demonstrates that the donor modification state can be inhibitory, but it did not identify the causal restriction enzyme and should therefore be interpreted as a Type-IV-like or modification-dependent barrier phenotype rather than a biochemically characterized Type IV system. A useful diagnostic comparison usually is to transform the same DNA sequence prepared in defined donor modification states while controlling topology, purity, and DNA quantity. Rescue by methylation-deficient DNA supports modification-dependent restriction, but causal assignment still requires identification and perturbation of the responsible locus. Failure in both donor states can indicate missing recipient-like protection or an unrelated delivery or replication problem.

A comparative overview of the four classical R-M mechanisms and representative enzyme structures is shown in Figure 1 below. For visual clarity, the schematic emphasizes recognition, methylation/protection, and cleavage logic rather than displaying a complete cofactor inventory; the relevant energetic and catalytic requirements are described in the corresponding subsections.

### 3.5. Atypical, Hybrid, and Other DNA-Modification-Associated Defense Systems

Not every methylation-associated antiphage locus fits neatly into the canonical four-class scheme. LlaI, encoded by the conjugative plasmid pTR2030 of *Lactococcus lactis*, is an atypical restriction-modification system containing a functional Type IIS-like methyltransferase, LlaIM, and three restriction-associated genes, *llaI.1*, *llaI.2*, and *llaI.3*. Genetic disruption of *llaI.1* abolished restriction, whereas disruption of *llaI.2* or *llaI.3* reduced restriction efficiency. The LlaI.2 protein contains nucleotide-binding motifs resembling those found in GTP-binding proteins, suggesting mechanistic features beyond a conventional Type II restriction endonuclease. However, this sequence evidence does not establish a canonical Type I ATP-dependent DNA-translocation mechanism. LlaI is therefore best regarded as an experimentally characterized atypical or hybrid R-M system rather than a conventional HsdRMS Type I complex [35].

BREX and DISARM should also be distinguished from classical R-M systems. BREX was originally characterized as a multicomponent phage-exclusion system in which sequence-specific host DNA methylation contributes to self/non-self discrimination without conventional cognate restriction [36]. Importantly for LAB, Hui et al. functionally characterized a BREX locus in *Lactobacillus casei* Zhang and demonstrated that PglX-dependent methylation was essential for phage resistance [37]. However, DNA methylation is not universal across BREX subtypes. Type-IV BREX replaces the canonical methyltransferase module with phosphorothioation-associated machinery, as supported by comparative analyses and experimental demonstration of DNA phosphorothioation in *Bacteroides salyersiae* [38,39]. This BREX subtype is distinct from classical Type-IV modification-dependent restriction systems. DISARM similarly combines DNA modification with helicase- and nuclease-associated components but operates through a broader defense mechanism than a conventional R-M pair [40].

Additional DNA modification-based defense mechanisms include Dnd- and Ssp-associated pathways, which introduce phosphorothioate modifications into the DNA backbone rather than methyl groups onto nucleobases [39]. Conversely, phages can employ noncanonical DNA modifications to counter host restriction. For example, Hutinet et al. demonstrated that 7-deazaguanine derivatives incorporated into phage DNA can confer resistance to selected restriction endonucleases [41]. These mechanisms extend beyond the classical Type I–IV R-M classification and were not specifically evaluated in our comparative genomic survey. Their occurrence and functional significance in LAB should not be inferred solely from observations in other bacterial hosts. A few selected R-M systems which have been experimentally characterized or otherwise supported previously are presented in Table 1 below.

Other antiphage systems, including CBASS, Thoeris, Zorya, Septu, Gabija, and retrons, can occur in defense islands together with R-M loci [7,8,42]. Genomic proximity may reflect a defense-rich mobile region but does not demonstrate functional cooperation. Throughout this review, the term defense repertoire is used for the set of antiphage systems encoded by a strain, but functional interactions still require matched experimental testing.

## 4. Regulation, Mobility, and Evolution of R-M Systems

An R-M system must protect newly replicated host DNA while retaining sufficient restriction activity against incoming DNA. Inadequate or delayed methylation can expose the chromosome to self-restriction, whereas weak restriction reduces defense. This balance is particularly important after horizontal acquisition of a new R-M locus, during replication-associated hemimethylation, or after loss of an R-M-bearing plasmid [21,43].

Some Type II systems use dedicated controller (C) proteins to delay restriction relative to methylation, allowing the newly acquired host genome to become protected before high nuclease activity is established. Promoter architecture, transcriptional coupling, gene dosage, and protein stability can also contribute to temporal regulation [44]. A nearby C-like open reading frame is therefore suggestive only when sequence, regulatory, or functional evidence supports that role.

Orphan MTases require a different interpretation. They may be remnants of decayed R-M systems, independently mobile enzymes, or regulatory methyltransferases with no active restriction partner. DNA methylation can influence replication, repair, chromosome organization, and transcription, but such functions cannot be assigned to an individual LAB MTase from annotation alone [45].

Mobility is central to R-M evolution in LAB. R-M loci occur on chromosomes, plasmids, and integrative elements, and acquisition of a mobile locus can change both phage susceptibility and DNA-transfer compatibility in a single step. This creates an evolutionary paradox: R-M systems can suppress horizontal gene transfer while themselves behaving as mobile genetic elements. Once established, post-segregational killing or related addiction-like effects can favor persistence of the R-M-bearing replicon because residual restriction activity becomes harmful after protective methylation is lost [21,44]. Lactococcal plasmids provide especially clear examples of R-M determinants embedded within broader collections of metabolic and antiphage functions [46,47]. Turnover need not remove an R-M system cleanly. Loss or inactivation of restriction activity while methylation persists can leave an orphan MTase, whereas recombination or acquisition of a new specificity determinant can redraw DNA-transfer compatibility without any corresponding change in the host core genome [21,22,45,48]. In evolutionary terms, the R-M repertoire can therefore change on a much shorter timescale than the host lineage itself—two closely related strains may differ sharply in restriction phenotype, while distantly related strains may carry superficially similar R-M classes for entirely different historical reasons.

At population scale, R-M systems can channel rather than simply suppress horizontal gene transfer. Exchange is favored among donors and recipients with compatible modification patterns and disfavored across incompatible combinations; over time, this can reshape plasmid distribution and lineage-specific gene flow [22,48]. Recognition-site avoidance in bacterial chromosomes, mobile elements, and phages provides another potential signature of sustained restriction pressure [10,49]. However, gain, loss, decay, and horizontal transfer can all produce patchy distributions. Host-tree/R-M incongruence is therefore expected, but by itself it is not evidence for a particular horizontal-transfer event. The evolutionary relationships among R-M components cannot be inferred reliably from broad system-type assignments alone. Homologous restriction or methylation proteins may represent vertically inherited orthologs, duplicated paralogs, or independently acquired homologs, while recombination and domain exchange can generate new recognition specificities without replacing the entire locus. In LAB, the mobility of plasmid-associated R-M systems and specificity components provides a plausible mechanism for rapid diversification of restriction barriers. Distinguishing these alternatives requires gene-family phylogenies, conserved gene-neighborhood comparisons, and assessment of the corresponding chromosome or plasmid context. Importantly, the evidentiary strength of evolutionary assignments differs among sequence-based predictions, methylome-supported enzyme assignments, and experimentally characterized restriction systems [21,22,26,46,47,48].

## 5. Diversity and Phylogenetic Distribution of R-M Systems in LAB

### 5.1. Evidence from Characterized LAB Systems and Genome Surveys

R-M loci are widespread but unevenly distributed among strains, replicons, and ecological lineages [22,24]. In LAB, this variation can alter plasmid compatibility, phage host range, and survival of cloning vectors even among closely related strains. Apparent locus counts also depend on assembly continuity, annotation rules, and whether orphan, partial, or ambiguous systems are counted, making strain-resolved and evidence-graded inventories more informative than unqualified genus-wide totals.

*Lactococcus lactis* and *Lactococcus cremoris* provide the largest body of direct evidence—characterized systems include Type II LlaAI, LlaBI, LlaDCHI, LlaDII, and LlaJI; Type III LlaFI; Type I HsdS specificity variants; and atypical LlaI [18,20,26,28,29,30,31,35]. Genome-scale studies further show that lactococcal mobile elements carry extensive strain-variable antiphage repertoires enriched in R-M systems [46,47]. In *S. thermophilus*, R-M coexists with one of the best-characterized LAB CRISPR-Cas systems. Sth455I and ICESt1-encoded Sth368I are direct Type II examples [32,33], whereas genome-scale surveys reveal marked strain-to-strain variation in broader antiphage repertoires [50]. In *L. plantarum*, by contrast, one of the most informative engineering observations is the strong dependence of transformation on donor methylation state [12]. Long-read studies in lactobacilli have also demonstrated species- and strain-specific methylation patterns [51]. For many other LAB genera, predicted R-M loci greatly outnumber systems with demonstrated motifs or phenotypes.

The imbalance in experimental evidence is itself important—*Lactococcus* and *S. thermophilus* have been studied intensively because of their industrial importance, tractability, and long history in dairy phage research, whereas many other LAB genera entered the era of genome-scale annotation before comparable mechanistic work existed. Consequently, a genus with few named R-M systems should not automatically be interpreted as R-M-poor: it may simply be experimentally under-characterized. Genome surveys and methylome studies broaden the taxonomic view, but they also widen the gap between “system detected” and “system understood” [46,47,50,51].

### 5.2. Original Comparative Phylogenomic Survey of 196 LAB Genomes

To complement the literature synthesis, we performed an original comparative genomic survey using a purposive, diversity-oriented panel of 196 RefSeq assemblies from ten LAB groups: *Lacticaseibacillus*, *Lactiplantibacillus*, *Lactobacillus*, *Lactococcus*, *Leuconostoc*, *Limosilactobacillus*, *Oenococcus*, *Pediococcus*, *Streptococcus*, and *Weissella*. Genera were selected to span phylogenetically diverse food- and biotechnology-associated LAB, and genome selection emphasized species/strain diversity while maintaining approximately balanced representation among groups. *Enterococcus* was excluded a priori to keep the comparative scope focused; this reflects study design and the distinct safety/clinical context of enterococci, not exclusion from the LAB concept [5]. The resulting panel contains 15–23 genomes per group and 110 species-level operational designations, but its taxonomic depth is deliberately uneven. All 23 *Streptococcus* genomes are *S. thermophilus*, a major industrial dairy starter and the streptococcal lineage in which strain-level phage-defense diversity is directly relevant to fermentation [50,52]. Thus, the *Streptococcus* arm functions primarily as an intraspecific survey, whereas several other genera contribute much broader interspecific diversity. The panel is intended for comparative phylogenomics rather than population-prevalence estimation. Detailed selection criteria, accession-level metadata, and computational methods are provided in Supplementary Methods and Supplementary Table S1. The analysis included assembly-associated plasmid sequences where available in the selected RefSeq records. Overall, 151 annotated plasmid replicons were represented across 73 assemblies. Plasmids not included in the deposited assemblies were outside the scope of the survey; therefore, the resulting R-M inventory may underestimate plasmid-associated system diversity.

The host phylogeny was inferred independently of R-M content from 100 curated conserved protein markers (17,456 aligned amino-acid positions) using partition-aware IQ-TREE 3.1.4 with ModelFinder v3, 1000 ultrafast bootstrap replicates, and 1000 SH-aLRT replicates. All 196 genomes were retained. Independently, R-M loci were screened with PADLOC 2.0.0 and DefenseFinder 3.0.0 and reconciled at the physical-locus level with explicit handling of partial and uncertain architectures. Candidate R-M loci identified by either detector were considered, and agreement between both programs was not required for inclusion. Overlapping predictions were reconciled using genomic coordinates, gene content, and system architecture. Of the 436 candidate physical loci, 213 were identified by both detectors, 100 by PADLOC only, and 123 by DefenseFinder only. These loci were subsequently classified according to type-specific completeness criteria, with incomplete or uncertain architectures retained separately. The final curation contained 417 complete predicted systems, 17 partial candidates, and two uncertain candidates; only two of the 784 genome-by-type state cells remained unresolved. A complete predicted system was defined by a type-compatible genetic architecture: restriction, methylation, and specificity functions for Type I; compatible restriction and methylation functions or a fused Type IIG architecture for Type II; Mod and Res functions for Type III; and a supported modification-dependent restriction architecture for Type IV, which does not require a cognate methyltransferase. These classifications represent computational predictions of architectural completeness and do not establish enzymatic activity or a restriction phenotype. The tree and R-M matrix were joined only after both analyses had been frozen independently. Full software versions, filters, curation rules, and validation procedures are described in Supplementary Methods; the deposited data and scripts are identified in the Data Availability Statement.

**Figure 2:**
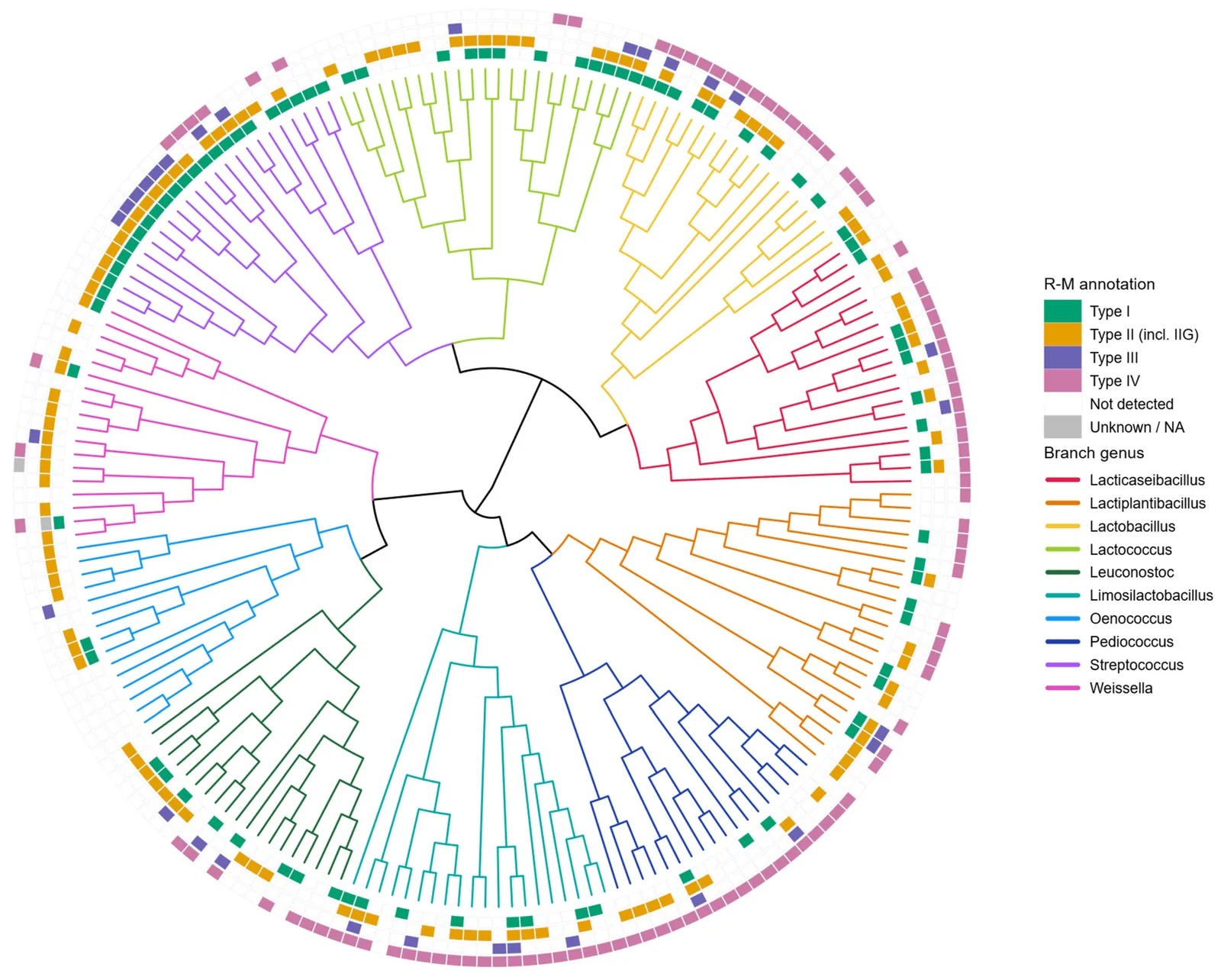
Phylogenetic distribution of predicted Type I–IV restriction-modification systems across the 196-genome LAB panel. The host phylogeny was inferred from 100 conserved protein markers. Branches are colored by operational genus where descendant assignments are homogeneous; mixed-genus backbone branches remain neutral. Four external rings indicate Type I, Type II (including IIG), Type III, and Type IV states from the independently curated R-M matrix. Saturated ring colors indicate complete predicted systems, white indicates successful non-detection, and grey indicates unresolved/NA states. The displayed tree is midpoint-rooted solely for visualization; evolutionary interpretation is based on the accepted unrooted topology. Detailed methods, accession-level states, and the unrooted phylogram are provided in the Supplementary Materials.

The host tree recovered each of the ten operational genera as a coherent unrooted split with 100/100 SH-aLRT/UFBoot support, supporting its use as a scaffold for comparative R-M mapping. That genus-level coherence should not be conflated with uniformly resolved deep history: one major backbone split received only 60.2/77 SH-aLRT/UFBoot support. We therefore use the tree primarily to compare R-M patterns among sampled lineages rather than to infer a directional sequence of early intergeneric divergences. Because no validated outgroup was included, the accepted phylogeny is explicitly unrooted. Figure 2 uses midpoint rooting only as a display convention. No ancestral or derived interpretation is assigned to that root. An unrooted square-root-compressed phylogram is provided as Supplementary Figure S1 to retain the original topology while displaying relative branch-length differences more clearly; the square-root transformation in that figure is graphical and should not be interpreted as an untransformed substitutions-per-site scale.

Across the panel, complete predicted Type II systems (including IIG) were present in 112 of 195 evaluable genomes, Type IV systems in 100 of 195, Type I systems in 86 of 196, and Type III systems in 30 of 196. The denominator of 195 for Types II and IV reflects one unresolved classification for each type: GCF_001932615.1 (*Weissella jogaejeotgali*) for Type II and GCF_041281755.1 (*Weissella sagaensis*) for Type IV. Both cases involved HNH-associated candidates whose broad-type assignments could not be resolved confidently. All 196 genomes were retained in the analysis, and unresolved states were not treated as confirmed absences. The distribution was strongly heterogeneous across the sampled lineages. Type IV calls occurred in all 18 sampled *Pediococcus*, 19/20 *Limosilactobacillus*, 17/19 *Lactobacillus*, and 16/19 *Lacticaseibacillus* genomes, but in none of the 15 *Oenococcus* genomes and only 2/23 *Lactococcus* genomes. Type I was especially common in the *S. thermophilus* panel (22/23 genomes), whereas Type II was more broadly distributed and Type III was comparatively sparse. These frequencies describe the curated comparative panel and should not be interpreted as genus-wide population prevalences. These counts also need to be interpreted at the scale represented by each branch. For *S. thermophilus*, 22/23 Type I-positive genomes indicate striking strain-level consistency within one species; the same fraction in a genus spanning many species would describe a different evolutionary pattern. Approximate balance in genome number reduces domination by heavily sequenced taxa, but it does not make the ten groups statistically equivalent samples of their genera. For this reason, Figure 2 is most informative as a comparison of phylogenetic pattern and lineage association rather than as a census of LAB prevalence.

To evaluate whether predicted R-M system distributions exhibited phylogenetic clustering, we performed exploratory parsimony-based character-randomization tests on the accepted 196-genome host tree (Supplementary Methods S7; Supplementary Tables S2 and S3) [53]. All four types exhibited lower minimum-change scores than expected under global tip-label randomization (FDR q ≤ 0.02449). Under a more restrictive null model preserving type-specific frequencies within operational genera, additional clustering was detected for Type II (q = 0.00032) and Type IV (q = 0.00508), but not for Type I (q = 0.487); Type III was borderline (q = 0.04939). Alternative assignments of the two unresolved classification states did not alter the conclusions for Types II and IV. However, a sensitivity analysis retaining one genome per species consistently supported global clustering only for Type IV, while within-genus significance was not consistently retained. Thus, the data support lineage-associated differences in predicted R-M repertoires within this comparative panel, but finer-scale signal is sensitive to species/strain representation, and the analyses cannot identify the evolutionary processes underlying the observed distributions.

The descriptive differences among R-M classes are notable within the sampled panel. Type IV predictions occur frequently in several sampled groups but are absent or rare in others. This pattern is compatible with lineage-associated differences in R-M repertoire composition; however, the permutation tests support clustering within this panel, but the species-balanced sensitivity analysis limits the robustness of finer-scale clustering, and neither analysis identifies the evolutionary mechanisms responsible for these differences. Type II, in contrast, is widespread across the panel but patchier within major groups, consistent with a large and mobile mechanistic class that can turn over without closely tracking genus boundaries. Several evolutionary histories could generate these patterns—long-term vertical persistence, recurrent loss, repeated acquisition, or differences in local phage selection—and the present host tree cannot distinguish among them [10,21,22,48]. The observed distribution motivates hypotheses of lineage-associated conservation and differential R-M repertoire turnover, but distinguishing these possibilities from independent acquisition, recurrent loss, or sampling effects requires additional analyses. Conversely, conservation of a broad R-M type does not imply conservation of its recognition sequence, gene architecture, or evolutionary origin; resolving these alternatives would require locus-level phylogenies, gene-content and synteny comparisons, and explicit analysis of plasmid or other mobile-element context. Those analyses are the appropriate route for testing individual horizontal-transfer events rather than inferring them from ring patterns alone.

## 6. Phage Resistance, Coevolution, and Interaction with Other Defenses

R-M systems act after phage DNA entry and can reduce efficiency of plaquing by several orders of magnitude before productive replication is established. Unlike CRISPR adaptation, R-M discrimination does not require prior acquisition of a phage-derived spacer; protection instead depends on recognition-site content, the modification state inherited from the previous host, R-M expression, and phage counter-defense [6,50,54]. The relevant identity of a phage is therefore partly epigenetic: the same genome sequence can encounter a different restriction barrier after propagation in a host that changes its modification pattern. R-M defense is consequently sensitive to infection history as well as genotype [9,10]. Phages can escape this pressure in mechanistically different ways: by mutating or depleting recognition sites, acquiring methyltransferases that reproduce a permissive modification state, replacing canonical bases with modified derivatives, encoding anti-restriction or DNA-mimic proteins, or otherwise reducing exposure of their genome to restriction [6,10,49,55]. These routes attack different parts of the same recognition problem: site loss changes sequence, methylation mimicry changes epigenetic state, and anti-restriction disables the host machinery directly. As a result, an R-M system that is highly effective against one phage population can lose effectiveness after repeated selection or passage through a permissive host without any change in the bacterial genotype.

A direct example of phage counter-defense in LAB comes from lactococcal 936-type (Skunavirus) phages encoding their own DNA methyltransferases. Murphy et al. identified phage-associated methyltransferases and used methylome analysis and heterologous expression to demonstrate protection against selected restriction enzymes, including HphI and DpnII [56]. These findings show that phage-encoded modification functions can counteract restriction and illustrate a molecular route by which LAB phages may escape host R-M barriers. Such protection remains dependent on the recognition specificity of the host restriction system and cannot be assumed to confer universal resistance.

Layered defenses can slow that escape because they interrogate different properties at different stages of infection, but complementarity is not automatic. In *S. thermophilus*, Dupuis et al. demonstrated that CRISPR-Cas and R-M can coexist and increase resistance when combined [57]. Genome surveys of *S. thermophilus* and lactococci likewise reveal extensive strain-level variation in broader defense repertoires [47,50]. Genomic co-occurrence alone, however, cannot establish synergy: combinations must be tested against the same phage under matched growth conditions. The useful question is therefore not simply how many defense systems a genome encodes, but which combination is active against the phage population currently encountered.

Industrial fermentation repeatedly selects both starters and their phages. Dense cultures amplify rare escape variants, while sanitation, strain rotation, receptor variation, R-M, CRISPR-Cas, and abortive infection impose different bottlenecks. Strain rotation can also change the modification history carried by phage progeny, meaning that two production hosts may differ not only in receptor compatibility but in the epigenetic state against which the next R-M barrier acts [9,10]. A single R-M system is therefore unlikely to provide indefinitely durable protection, and production-relevant phage control requires monitoring contemporary phage populations and evaluating layered defense combinations rather than relying on a single historical laboratory isolate [6,50,52].

## 7. Methylome Analysis and Modern Detection of LAB R-M Systems

Genome annotation can identify candidate REases and MTases, but it cannot determine whether they are expressed, active, or responsible for a biological phenotype. Native-DNA methylome analysis narrows this gap by revealing modified bases and enriched sequence contexts. It is especially useful in strains containing multiple partial, orphan, or plasmid-borne loci, provided that motif detection, enzyme assignment, and restriction phenotype remain separate evidentiary steps. Single-molecule real-time (SMRT) sequencing detects base modification through changes in polymerase kinetics and has enabled genome-wide discovery of bacterial methylation motifs, particularly N6-methyladenine (6mA) and N4-methylcytosine (4mC) [58,59,60]. Detection of 5-methylcytosine (5mC) is more demanding and depends on chemistry, depth, and analytical method. Long reads are particularly valuable for plasmid-rich LAB because they can simultaneously improve closure of chromosomes and accessory replicons while preserving native modification information.

Nanopore sequencing detects modifications directly from changes in ionic-current signal. Early demonstrations focused mainly on cytosine methylation [61,62], whereas current-generation models can detect bacterial 6mA, 4mC, and 5mC. Recent benchmarking nevertheless shows substantial dependence on modification context, neighboring methylated bases, read quality, coverage, basecalling model, and analysis pipeline [63]. Modification probabilities should therefore not be compared uncritically across runs or software versions; unmethylated or whole-genome-amplified controls, biological replication, motif-level aggregation, and orthogonal validation remain important, especially for non-canonical or partially methylated motifs. Assigning a motif to a particular enzyme is often the difficult step because a strain may encode several related MTases, one enzyme may act on multiple motifs, a locus may be silent under the sequencing condition, or a high-copy plasmid may dominate the signal. Deletion or inactivation of the candidate gene, expression in a methylation-naive host, comparison with a plasmid-cured derivative, or purified-enzyme assays can distinguish these alternatives [51,64]. Motif detection establishes an active modification pattern, but it does not by itself prove that a neighboring restriction gene is intact or responsible for transformation failure.

From an evidentiary perspective, methylome analysis is best viewed as a causal ladder rather than an endpoint: modified base → recurrent motif → active MTase → assignment to a specific locus → demonstration that the same locus changes restriction or transformation. Each step answers a different question. A reproducible motif can establish methylation activity while saying nothing about whether a neighboring REase is expressed; conversely, a donor-methylation phenotype can reveal modification-sensitive restriction without identifying the enzyme responsible. Combining native methylome data with targeted genetics is therefore substantially more informative than either approach alone [51,58,59,60,61,62,63,64].

## 8. R-M Systems as Barriers to Genetic Engineering

Low transformation efficiency is a composite phenotype with mechanistically distinct causes, including inefficient DNA uptake, cell-envelope barriers, membrane injury, DNA topology, plasmid replication failure, insufficient selectable-marker expression, construct toxicity, and restriction of incoming DNA. Of these mechanisms, only restriction of DNA by an active R-M system constitutes an R-M-mediated transformation barrier. A causal role for R-M is supported when transformation changes in response to defined donor modification states, recognition-site content, or targeted alteration of a candidate restriction locus. The first useful comparison is often the same plasmid prepared in controlled methylation states. Conventional *E. coli* donors install Dam and Dcm methylation, whereas Dam-/Dcm- hosts do not introduce those modifications. In selected *L. plantarum* strains, non-methylated DNA markedly improved transformation [12]—this result makes methylation-deficient donors a useful diagnostic condition but still not a universal solution. The diagnostic logic matters because classical and Type-IV-like barriers can demand opposite epigenetic interventions—for Types I–III, incoming DNA may fail because it lacks recipient-compatible methylation, so cognate premodification can protect it; at the same time, for a modification-dependent Type-IV-like barrier, donor methylation itself can create the restriction signal, so removing common donor marks may improve survival [12,65]. Treating all poor transformation as a single “methylation problem” is therefore misleading: the same manipulation can rescue one strain and worsen or leave unchanged another.

A strain-level connection between genomic R-M prediction and genetic engineering is provided by *Lactococcus lactis* C20. This strain is represented in our comparative panel by assembly GCF_035985965.1, for which the curated analysis identified a complete predicted Type II/IIG system. Independently, Chen et al. demonstrated that donor premodification compatible with the recipient methylation pattern substantially improved plasmid transformation in C20 [14]. Their study also evaluated CRISPR-associated plasmids, illustrating that vectors intended for CRISPR-based editing remain subject to the recipient’s DNA-entry and restriction barriers. However, the co-occurrence of a predicted R-M system and a methylation-dependent transformation phenotype does not establish that the particular predicted locus was responsible. Such assignment would require locus-specific genetic or biochemical validation.

The mimicking-of-DNA-methylation-patterns (MoDMP) strategy established the protective side of this logic across difficult bacterial hosts [65]. In *L. lactis* C20, donor premodification improved plasmid transformation and, together with rolling-circle amplification in the reported workflow, yielded approximately 10^4^ transformants per microgram under the best tested condition [14]. Such rescue experiments are informative beyond optimization. When DNA sequence, amount, and replication competence are controlled, rescue by recipient-like premodification supports the conclusion that missing protective methylation contributed to the barrier; rescue by methylation-deficient donor DNA instead points toward modification-dependent restriction. In both cases, locus-level validation is still required to identify the causal enzyme.

Recognition-site depletion acts on the DNA sequence rather than its epigenetic state. Johnston et al. demonstrated systematic removal of restriction sites as a general strategy for evading bacterial R-M barriers [13]. This is most practical for synthetic operons, editing templates, and longer motifs; short frequent sites may be difficult to eliminate without perturbing codon use, RNA structure, regulatory elements, or assembly behavior. Sequence redesign and premodification are therefore complementary rather than competing approaches: redesign can remove avoidable sites, while methylation can protect those that cannot be changed.

Conjugation and transduction solve a different problem—DNA entry. Conjugative transfer from *E. coli* has enabled DNA delivery to transformation-resistant lactobacilli [66], while phage-mediated transfer has been demonstrated in lactococci and from *S. thermophilus* to *L. lactis* [67,68]. An increase in transformant recovery after changing the delivery route does not, by itself, show that restriction was bypassed; it may simply mean that an envelope or electroporation bottleneck was avoided. Delivery, restriction, replication, and selection should therefore be treated as separable experimental variables.

Recipient-side R-M inactivation is the most direct intervention but also the most biologically consequential. Selective loss of restriction while retaining protective methylation can, in principle, improve genetic accessibility and reduce the risk of self-cleavage during engineering, whereas complete curing or poorly ordered deletion can weaken phage defense, alter gene flow, or destabilize an addiction-like element [21,43]. For industrial strains, recipient modification is therefore better treated as a causal, last-resort intervention after donor-side approaches and barrier validation rather than as a default route to transformability.

Table 2 presents various strategies to overcome R-M associated barriers, but it should be read as a diagnostic map rather than a ranking of engineering methods. Each intervention also functions as a mechanistic experiment: a methylation-deficient donor tests whether common donor marks are harmful, cognate premodification tests whether recipient-like protection is missing, site depletion tests whether a defined recognition motif is limiting, and alternative delivery tests DNA entry—combining these interventions can be powerful, but doing so without this logic may improve recovery while obscuring why it worked.

## 9. Industrial and Biotechnological Implications

The most direct industrial value of R-M knowledge is improved management of starter-culture phage resistance. Resident or introduced R-M systems can reduce phage propagation, and lactococcal mobile elements provide a natural source of defense functions [46,47]. The objective, however, is not simply to maximize restriction. A useful defense must be established without autoimmunity, remain compatible with desirable plasmids and future engineering, and function under production conditions. Work in *S. thermophilus* indicates that complementary defense systems can strengthen resistance, but each combination must still be tested against a relevant phage panel [52,57]. In other words, the industrially useful phenotype is context-dependent resistance, not the largest possible number of defense loci.

LAB are also widely used as engineering hosts for production or delivery of bacteriocins, metabolites, enzymes, antigens, and recombinant proteins [3,4,69]. *L. lactis* is particularly tractable, whereas industrial isolates of lactobacilli and related genera can be substantially harder to manipulate. Resolving strain-specific R-M barriers allows engineering to remain in strains selected for fermentation performance, colonization, or metabolism rather than forcing desired functions into a small set of domesticated laboratory chassis. Permanent removal of R-M activity may nevertheless be undesirable. A more useful distinction is between the construction phase and the production phase: during strain construction, a validated barrier may be temporarily bypassed or selectively attenuated, while the finished production strain may benefit from retaining or restoring the defense state that protects it from phages. Donor-side strategies such as premodification, donor choice, or recognition-site redesign are attractive in this respect because they can improve DNA entry without permanently rewriting the recipient defense repertoire. Recording genome sequence, plasmid content, and methylome together would make both engineering and later interpretation of phage susceptibility more reproducible.

## 10. Conclusions and Future Perspectives

Restriction–modification systems connect phage defense, plasmid mobility, lineage-specific gene flow, and amenability to genetic manipulation in LAB. The strongest mechanistic evidence still comes from experimentally characterized lactococcal and streptococcal systems, while long-read methylomes and comparative genomics reveal many additional candidates whose restriction phenotypes remain untested. The 196-genome analysis presented here reinforces that distinction: predicted R-M systems occur unevenly among sampled lineages, with phylogenetic patterns sensitive to species/strain representation, and the broad distribution of predicted systems should not be equated with demonstrated activity.

The main unresolved problem for now is causality—a predicted locus may be associated with a methylation motif, yet considerably fewer studies show that the encoded restriction activity cleaves incoming DNA or changes phage susceptibility in its native host. Closing that gap requires targeted gene deletion or inactivation together with controlled transformation or infection experiments. Transformation data become mechanistically informative only when donor modification, recognition-site content, DNA entry, plasmid replication, and selection are separated. Engineering should therefore follow the barrier that has actually been demonstrated: methylation-deficient donors are appropriate when donor methylation is inhibitory, classical restriction is better addressed by cognate premodification or removal of recognition sites, conjugation can solve inefficient DNA entry, but not necessarily post-entry restriction, and recipient-side R-M editing is justified only for a validated causal target. Industrial defense repertoires should be altered as little as necessary because the same systems that obstruct strain construction may protect the finished production strain.

Future studies should combine closed chromosome and plasmid assemblies, native methylomes, curated defense-locus annotation, locus-specific perturbation, standardized transformation measurements, and phage testing under process-relevant conditions. Larger and more taxonomically balanced comparative panels will also be valuable for distinguishing genus-level patterns from species-specific or strain-specific effects. With these data, individual R-M systems can be evaluated in the context that matters most: whether they protect a production strain, obstruct useful DNA transfer, or do both.

## Figures and Tables

**Table 1:** Selected experimentally characterized or phenotype-supported R-M systems in lactic acid bacteria.

Example	Host	Class	Genetic Context	Experimentally Established Feature	Evidence Level	Key Reference(s)
HsdS specificity variants	*Lactococcus lactis*	Type I	HsdRMS specificity module	Target-recognition-domain exchange generated new sequence specificities	Direct genetic specificity reconstruction	[26]
LlaBI	*L. lactis* subsp. *cremoris* W56	Type II	Compact R-M locus	Recognition of 5′-CTRYAG-3′ and bacteriophage-resistance phenotype	Direct genetic and enzymatic evidence	[18,28]
LlaDII	*L. lactis* subsp. *cremoris* W39	Type II	Plasmid pHW393	Recognition/cleavage of 5′-GCNGC-3′ and restriction of 936, P335, and c2 phages	Direct biochemical and phage evidence	[30]
LlaJI	*Lactococcus lactis*	Type II-like multigene system	Plasmid pNP40	Two MTase and two REase genes required for full phenotype; phage resistance strongest at 19 °C	Direct functional and phage evidence	[31]
Sth368I	*Streptococcus thermophilus* CNRZ368	Type II	Integrative element ICESt1	Mobile chromosomal R-M system genetically characterized	Direct genetic characterization	[33]
LlaFI	*Lactococcus lactis* LL42-1	Type III	Plasmid pND801; adjacent mod/res	Type III architecture and characteristic ATP/Mg^2+^ cofactor dependence	Direct genetic and biochemical evidence	[20]
Donor-methylation-sensitive barrier	*Lactiplantibacillus plantarum* (selected strains)	Type-IV-like phenotype; enzyme unresolved	Recipient locus not assigned in the original study	Methylation-deficient donor DNA markedly increased transformation	Transformation phenotype only	[12]
LlaI	*Lactococcus lactis*	Atypical/hybrid R-M	Conjugative plasmid pTR2030	Type IIS-like MTase plus three genes required for in vivo restriction	Direct genetic evidence; atypical classification	[35]

**Table 2:** Strategies for overcoming R-M-associated barriers in LAB and the interpretation of each intervention.

Strategy	Barrier Addressed	Evidence in or Relevant to LAB	Valid Interpretation	Principal Limitations
Methylation-deficient donor DNA	Modification-dependent restriction of donor methylation	Non-methylated DNA markedly improved direct cloning in selected *L. plantarum* strains [12].	The tested donor methylation state is inhibitory in that strain/construct.	Not universal; does not provide recipient-like protective methylation and does not identify the causal enzyme by itself.
Cognate premodification	Type I–III restriction of DNA lacking host-like marks	MoDMP proof of principle [65]; direct premodification in *L. lactis* C20 [14].	Reproducing an active recipient methylation pattern can protect incoming DNA.	Requires correct motif/enzyme assignment; incomplete methylation, Type IV activity, construct effects, and donor toxicity can remain.
Restriction-site depletion	High burden of known cognate sites	Systematic bacterial proof of principle [13].	Fewer target sites can reduce the probability of restriction.	Short motifs may be difficult to remove; recoding can perturb expression, RNA structure, or regulatory elements.
Conjugative delivery	Inefficient uptake of purified DNA	Transfer from *E. coli* into transformation-resistant lactobacilli [66].	Conjugation can bypass an entry bottleneck and broaden accessible hosts.	Does not automatically evade R-M; host range, counterselection, replication, and regulatory constraints remain.
Phage-mediated transduction	DNA delivery through a compatible phage particle	PhiLC3 cos-plasmid transfer in *L. lactis*; interspecies plasmid transfer from *S. thermophilus* to *L. lactis* [67,68].	Phage packaging can deliver DNA to recipients that are difficult to transform directly.	Host range, packaging limits, phage safety, and recipient defense constrain use; R-M can still act after injection.
Recipient R-M inactivation	Persistent validated recipient restriction activity	Conceptually justified after locus-specific causal validation; tractable laboratory chassis are not by themselves proof that R-M deletion caused transformability.	Removing a demonstrated restriction activity can improve accessibility if protective methylation and viability are preserved.	May weaken phage defense, alter gene flow or plasmid maintenance, and create autoimmunity if restriction/modification are altered in the wrong order.
Combined donor and vector redesign	Multiple simultaneous restriction/replication barriers	Supported by complementary evidence for premodification and site depletion [13,14,65].	Combined interventions are rational when independent barriers have been demonstrated.	More laborious; each component requires controls, and transformant recovery alone does not identify the limiting step.

## Data Availability

The genomic metadata, conserved-marker alignments, phylogenetic trees, restriction–modification system annotations, native detector outputs, statistical analyses, and associated reproducibility scripts generated in this study are available through Zenodo at https://doi.org/10.5281/zenodo.23888771. The original genome assemblies can be retrieved independently from NCBI RefSeq using the exact accession identifiers provided in Supplementary Table S1. Supplementary Methods and accession-level R-M states are provided with this article.

## Supplementary Materials

The supporting information can be downloaded at https://www.mdpi.com/article/10.3390/ijms272110007/s1.
