# Supplementary material for: Restriction–Modification Systems in Lactic Acid Bacteria: Diversity, Mechanisms, Evolution, and Biotechnological Applications
Source: Int J Mol Sci. 2026 Nov 8;27(21):10007. doi: 10.3390/ijms272110007 (PMC13764132; doi:10.3390/ijms272110007)
Supplement: Supplementary file 1 [file ijms-27-10007-s001.zip › ijms-4662330-supplementary.pdf]

Supplementary Materials

Restriction–Modification Systems in Lactic Acid Bacteria: Diversity, Mechanisms, Evolution, and Biotechnological Applications

Supplementary Methods

S1. Study design and genome panel

The comparative analysis used a purposive, diversity-oriented panel intended to span phylogenetically diverse LAB with food-fermentation or biotechnology relevance while avoiding domination by heavily sequenced taxa. Ten operational groups were included: Lacticaseibacillus, Lactiplantibacillus, Lactobacillus, Lactococcus, Leuconostoc, Limosilactobacillus, Oenococcus, Pediococcus, Streptococcus, and Weissella. Enterococcus was excluded a priori as a study-scope decision. The final panel contained 196 exact-version RefSeq GCF accessions and 110 species-level operational designations, with 15–23 assemblies per operational group. Streptococcus was intentionally restricted to S. thermophilus, the established industrial streptococcal starter lineage represented in this study; consequently, results for this operational group describe strain-level diversity within S. thermophilus and should not be generalized to the genus Streptococcus as a whole. The panel should therefore be interpreted as a diversity-oriented comparative dataset rather than a prevalence-weighted sample of LAB diversity.

The accepted panel was assembled from an original 177-genome selection plus 19 strict-valid historical anchor additions. Assembly-level composition was 150 Complete Genome, 5 Chromosome, and 41 Scaffold records. Reported screening criteria included a current exact accession, a ranked genus and species-level taxonomic designation, no Enterococcus ancestry, assembly level Complete Genome/Chromosome/Scaffold, reported contig count <=100, reported CheckM completeness >=95%, and reported contamination <=5%. Exact accessions and genome-level R-M states are provided in Supplementary Table S1.

S2. Sequence acquisition and validation

All 196 exact-version assemblies were retrieved with NCBI Datasets CLI 18.38.0. Genome FASTA, protein FASTA, CDS nucleotide sequences, GFF3, GenBank flat files, and NCBI sequence reports were retained. Independent validation checked package membership and checksums, genomic record identities and lengths, CDS/protein/GFF/GBFF joins, source translations, locus identity, and documented pseudogene/partial-translation exceptions. No approved genome was silently replaced or pruned.

The retrieved RefSeq assemblies included both chromosomal and assembly-associated plasmid sequences. The source-replicon inventory comprised 2,120 sequence records, including 151 records annotated as plasmids across 73 of the 196 assemblies. R-M detection used the annotated protein sequences of the selected assemblies, with replicon identities and genomic ordering preserved during input preparation and locus reconciliation. Plasmid-associated candidates were included in the curation process; notably, a Type I locus in *Leuconostoc mesenteroides* F17 (GCF_047344935.1) was accepted following validation of an origin-spanning architecture on circular plasmid NZ_CP178853.1. Plasmids not represented in the selected RefSeq assemblies were not independently retrieved. Consequently, the analysis does not constitute an exhaustive survey of all plasmids potentially carried by the sampled strains.

S3. Conserved host-marker selection

Conserved host proteins were identified with GToTree 1.8.10/HMMER 3.4 using a pinned 119-profile Firmicutes marker set. Stored profile GA thresholds were used. Multicopy ambiguity was handled by marking affected marker/genome cells missing rather than selecting an arbitrary best hit. The original frozen filtering retained 101 marker profiles. Independent orthology review identified two profile families (IPPT/PF01715 and IPT/PF01745) that selected the same complete MiaA protein at 192 exact source loci. The redundant IPT family was therefore excluded before alignment or topology inference, leaving 100 non-duplicated marker families and 19,359 accepted marker sequences. All 196 genomes passed the post-filter recovery criterion.

S4. Alignment and phylogenetic inference

Each of the 100 markers was aligned with pinned MAFFT and trimmed with trimAl. Independent validation reconstructed retained columns from source sequences and verified alignment formats, taxon identifiers, marker ordering, and partition coordinates. The primary concatenation contained all 196 genomes, 100 marker partitions, and 17,456 amino-acid positions. The accepted host phylogeny was inferred with IQ-TREE 3.1.4 using a partition-aware analysis, ModelFinder (MFP) separately across marker partitions without partition merging, 1,000 ultrafast bootstrap replicates, 1,000 SH-aLRT replicates, seed 1961008, two threads, and -keep-ident so that all exact accession labels remained in the tree. The final tree contains all 196 tips, finite non-negative branch lengths,

and paired SH-aLRT/UFBoot labels for 193 internal edges. The host-marker alignment contains 182 distinct full aligned strings and eight exact-identity groups comprising 22 taxa. Internal ordering within an exact-identity group is not resolved by these marker characters even if a bifurcation is displayed.

No outgroup was retrieved and validated for the accepted analysis; the inferential tree is therefore unrooted. The primary manuscript rendering applies midpoint rooting only for graphical layout. The supplementary unrooted phylogram retains the original unrooted topology, its branch radii are square-root transformed solely to improve visual separation of short branches. Consequently, displayed radial lengths are not an untransformed substitutions-per-site scale.

S5. R-M system detection and curation

R-M detection was performed independently of the host tree. PADLOC 2.0.0 (PADLOC-DB 2.0.0) and DefenseFinder 3.0.0 (DefenseFinder models 3.0.0; MacSyFinder 2.1.4; HMMER 3.4) were run for all 196 assemblies. Replicon identity, gene order, locus tags, protein accessions, strand, and coordinates were retained throughout. DefenseFinder inputs were reconstructed with unique generated positional identifiers so that repeated WP accessions did not collapse physically distinct loci.

PADLOC produced 313 raw system calls. DefenseFinder produced 382 R-M-like rows; after literal duplicate removal and reconciliation of overlapping same-type model solutions, these represented 336 physical DefenseFinder loci. Cross-detector physical-locus reconciliation yielded 213 shared PADLOC/DefenseFinder loci, 100 PADLOC-only loci, and 123 DefenseFinder-only loci, for 436 candidate physical loci in total. Broad-type conflicts were absent among shared loci.

Curation distinguished COMPLETE_PREDICTED, PARTIAL_CANDIDATE, UNCERTAIN, successful non-detection, and unresolved/NA states. Type I required compatible R/M/S functionality or equivalent supported architecture; incomplete R+M, R+S, or M+S configurations remained partial unless local evidence rescued the missing component. Ordinary Type II systems required compatible restriction/methylation architecture; fused Type IIG/IIG_2 systems were accepted and collapsed into broad Type II for the four-ring analysis. Type III required compatible Mod/Res architecture. Type IV modification-dependent restriction systems could be single-component and did not require a cognate MTase. Two HNH nuclease candidates remained unresolved rather than being forced into Type II or Type IV.

Final curation yielded 436 candidate loci: 417 COMPLETE_PREDICTED, 17 PARTIAL_CANDIDATE, and 2 UNCERTAIN. Accepted complete systems comprised 101 Type I, 162 Type II including IIG, 33 Type III, and 121 Type IV systems. Genome-level positive calls were 86 Type I, 112 Type II, 30 Type III, and 100 Type IV. The final 196 × 4 state matrix contained 328 cells with at least one complete predicted system, 442 cells with no detected system after successful screening, 12 cells containing partial candidates but no accepted complete system, and two unresolved/NA cells. The 12 partial-only cells were encoded as zero in the primary binary visualization but remained distinguishable in the full curation table.

The original per-assembly PADLOC and DefenseFinder outputs, including system-level, gene-level and HMM-profile evidence, as well as the positional identifier mappings used for physical-locus reconciliation, are provided in the associated Zenodo dataset. The deposit also contains normalized detector-call tables, the curated 436-locus inventory, processing scripts, source assembly accession lists, and source-sequence integrity records. Software and model database versions are specified in the accompanying metadata.

S6. Tree-R-M integration and visualization

The accepted Stage-4 host tree and frozen Stage-5 R-M matrix were joined by exact versioned GCF accession. Join validation confirmed 196 unique tree tips, 196 unique R-M rows, 784 genome-by-type cells, no missing accessions, no duplicate accessions, and zero consistency errors. Type I, Type II including IIG, Type III, and Type IV are displayed as four concentric rings. Accepted complete systems are shown with type-specific colors, successful non-detection as white, and unresolved/NA as grey. Branches receive a genus color only when all descendant tips share the same operational genus; mixed-genus backbone branches are neutral.

Supplementary Figure S1

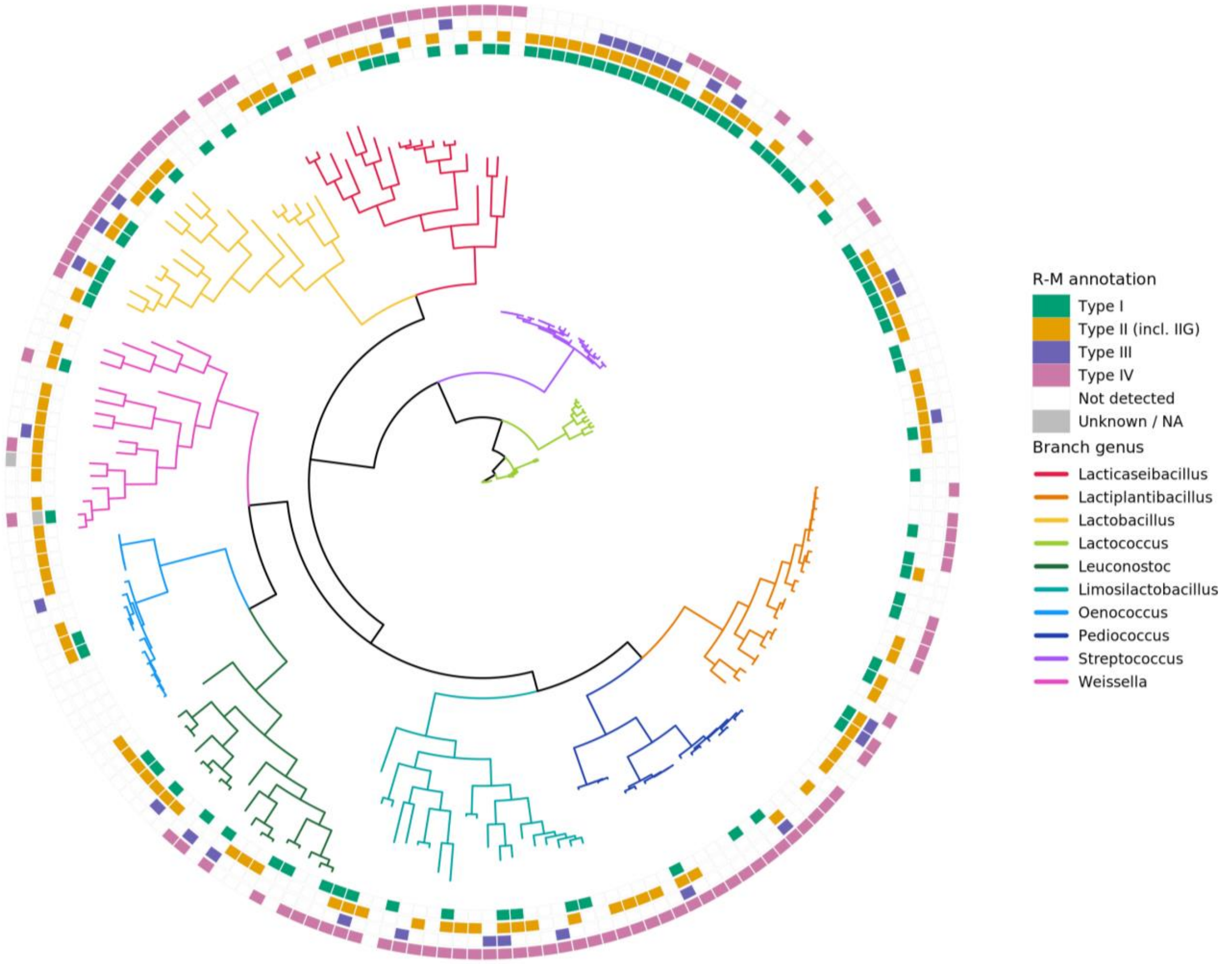

Supplementary Figure S1. Unrooted host phylogeny of the 196-genome LAB panel with Type I–IV R-M annotation rings. The accepted unrooted 100-marker IQ-TREE topology is shown using a square-root-compressed branch-radius display to improve visibility of short and long branches in the same circular layout. The square-root transformation is graphical only; topology and support values are unchanged, and radial lengths are not an untransformed substitutions-per-site scale. Four rings show Type I, Type II including IIG, Type III, and Type IV states using the same state definitions and palette as Figure 2 of the main manuscript.

Supplementary Table S1. Genome-level distribution and curation of restriction-modification systems in the approved 196-genome LAB panel

Each R-M type cell is reported as count / curation state / primary-figure state. C = COMPLETE_PREDICTED; ND = NOT_DETECTED_AFTER_SUCCESSFUL_SEARCH; P = PARTIAL_CANDIDATE; U = UNCERTAIN. Primary-figure states: 1 = at least one accepted system; 0 = successful screen with no accepted system; NA = unresolved. Type IIG is retained as a subtype but collapsed into broad Type II for the main figure. Full locus identifiers, detector-level evidence, and reproducibility scripts are included in the accompanying Zenodo dataset.

Assembly accession	Organism	Strain / isolate	Figure genus	Type I count/state/fig	Type II count/state/fig	Type III count/state/fig	Type IV count/state/fig	Accepted subtypes	Curation exceptions
**GCF_000009425.1**	Lactococcus cremoris subsp. cremoris MG1363	MG1363	Lactococcus	1 / C / 1	0 / ND / 0	0 / ND / 0	0 / ND / 0	I: RM_type_I;RM_Type_I	
**GCF_000010145.1**	Limosilactobacillus fermentum IFO 3956	IFO 3956	Limosilactobacillus	0 / ND / 0	0 / ND / 0	1 / C / 1	1 / C / 1	III: RM_type_III;RM_Type_III<br>IV: RM_type_IV	
**GCF_000011045.1**	Lacticaseibacillus rhamnosus GG	ATCC 53103	Lacticaseibacillus	0 / ND / 0	0 / ND / 0	1 / C / 1	2 / C / 1	III: RM_type_III;N6_N4_RM_Type_III<br>IV: GmrSD_RM_Type_IV | RM_Type_IV	
**GCF_000014385.1**	Oenococcus oeni PSU-1	PSU-1	Oenococcus	0 / ND / 0	0 / ND / 0	0 / ND / 0	0 / ND / 0		
**GCF_000014425.1**	Lactobacillus gasseri ATCC 33323 = JCM 1131	ATCC 33323	Lactobacillus	1 / C / 1	0 / ND / 0	0 / ND / 0	2 / C / 1	I: RM_type_I;RM_Type_I<br>IV: GmrSD_RM_Type_IV | RM_type_IV	
**GCF_000014445.1**	Leuconostoc mesenteroides subsp. mesenteroides ATCC 8293	ATCC 8293	Leuconostoc	0 / ND / 0	0 / ND / 0	0 / ND / 0	1 / C / 1	IV: RM_Type_IV	
**GCF_000014505.1**	Pediococcus pentosaceus ATCC 25745	ATCC 25745	Pediococcus	0 / ND / 0	1 / C / 1	0 / ND / 0	1 / C / 1	II: RM_type_II;DNA_met_RM_Type_II_PDDEXK<br>IV: RM_type_IV	
**GCF_000014545.1**	Lactococcus cremoris subsp. cremoris SK11	SK11	Lactococcus	0 / ND / 0	2 / C / 1	1 / C / 1	0 / ND / 0	II: RM_type_IIG;RM_Type_IIG | RM_type_IIG;RM_Type_IIG_2<br>III: RM_type_III;RM_Type_III	
**GCF_000015385.1**	Lactobacillus helveticus DPC 4571	DPC 4571	Lactobacillus	1 / C / 1	1 / C / 1	1 / C / 1	2 / C / 1	I: RM_type_I;N6_RM_Type_I<br>II: RM_type_IIG;RM_Type_IIG_2<br>III: RM_type_III;RM_Type_III<br>IV: RM_type_IV	SHARED:GCF_000015385.1:7|Type_I|PARTIAL_CANDI<br>DATE|RM_type_I;RM_Type_I
**GCF_000016825.1**	Limosilactobacillus reuteri subsp. reuteri JCM 1112 = DSM 20016	DSM 20016	Limosilactobacillus	2 / C / 1	0 / ND / 0	0 / ND / 0	1 / C / 1	I: RM_type_I;N6_RM_Type_I | RM_type_I;RM_Type_I<br>IV: RM_type_IV	
**GCF_000023085.1**	Lactiplantibacillus plantarum JDM1	JDM1	Lactiplantibacillus	0 / ND / 0	0 / ND / 0	0 / ND / 0	1 / C / 1	IV: RM_Type_IV	
**GCF_000025045.1**	Lactococcus lactis subsp. lactis KF147	KF147	Lactococcus	0 / ND / 0	2 / C / 1	0 / ND / 0	0 / ND / 0	II: RM_type_II | RM_type_IIG;RM_Type_IIG	
**GCF_000026405.1**	Leuconostoc citreum KM20	KM20	Leuconostoc	1 / C / 1	0 / ND / 0	0 / ND / 0	1 / C / 1	I: RM_type_I;N6_RM_Type_I<br>IV: RM_type_IV	
**GCF_000092505.1**	Leuconostoc kimchii IMSNU 11154	IMSNU 11154	Leuconostoc	1 / C / 1	0 / ND / 0	3 / C / 1	1 / C / 1	I: RM_type_I;RM_Type_I<br>III: RM_type_III;N6_N4_RM_Type_III | RM_type_III;RM_Type_III<br>IV: RM_type_IV	
**GCF_000148815.2**	Lactiplantibacillus plantarum ST-III	ST-III	Lactiplantibacillus	0 / P / 0	0 / ND / 0	0 / ND / 0	0 / ND / 0		DFONLY:GCF_000148815.2:001|Type_I|PARTIAL_CAN<br>DIDATE|N6_RM_Type_I
**GCF_000155515.2**	Lacticaseibacillus paracasei subsp. paracasei 8700:2	362.5013889	Lacticaseibacillus	1 / C / 1	0 / ND / 0	0 / ND / 0	3 / C / 1	I: RM_type_I;RM_Type_I<br>IV: GmrSD_RM_Type_IV | RM_Type_IV	
**GCF_000160835.1**	Limosilactobacillus antri DSM 16041	DSM 16041	Limosilactobacillus	1 / C / 1	1 / C / 1	1 / C / 1	1 / C / 1	I: RM_type_I;RM_Type_I<br>II: RM_type_II<br>III: RM_type_III;RM_Type_III<br>IV: RM_type_IV	
**GCF_000203855.3**	Lactiplantibacillus plantarum WCFS1	WCFS1<br>NCIMB8826	Lactiplantibacillus	1 / C / 1	0 / ND / 0	0 / ND / 0	1 / C / 1	I: RM_type_I;RM_Type_I<br>IV: RM_Type_IV	
**GCF_000237995.1**	Pediococcus claussenii ATCC BAA-344	ATCC BAA-344	Pediococcus	0 / P / 0	1 / C / 1	0 / ND / 0	1 / C / 1	II: RM_type_II<br>IV: RM_type_IV	DFONLY:GCF_000237995.1:001|Type_I|PARTIAL_CAN<br>DIDATE|RM_Type_I
**GCF_000241055.1**	Oenococcus kitaharae DSM 17330	DSM 17330	Oenococcus	0 / ND / 0	2 / C / 1	0 / ND / 0	0 / ND / 0	II: RM_type_II;RM_Type_II | RM_type_IIG;RM_Type_IIG	
**GCF_000269925.1**	Lactococcus garvieae ATCC 49156	ATCC 49156	Lactococcus	0 / ND / 0	0 / ND / 0	0 / ND / 0	1 / C / 1	IV: RM_Type_IV	
**GCF_000269945.1**	Lactococcus garvieae Lg2	Lg2	Lactococcus	0 / ND / 0	0 / ND / 0	0 / ND / 0	1 / C / 1	IV: RM_Type_IV	
**GCF_000298875.1**	Leuconostoc gelidum JB7	JB7	Leuconostoc	0 / ND / 0	2 / C / 1	0 / ND / 0	0 / ND / 0	II: RM_Type_II | RM_type_II;D12_RM_Type_II_PDDEXK	
**GCF_000468955.1**	Lactococcus cremoris subsp. cremoris KW2	KW2	Lactococcus	1 / C / 1	2 / C / 1	0 / ND / 0	0 / ND / 0	I: RM_type_I;N6_RM_Type_I<br>II: RM_Type_II | RM_Type_IIG	
**GCF_000691805.2**	Weissella oryzae SG25	SG25	Weissella	1 / C / 1	2 / C / 1	0 / ND / 0	1 / C / 1	I: RM_type_I;N6_RM_Type_I<br>II:<br>RM_type_II;D12_RM_Type_II_PDDEXK;N6_N4_RM_Ty<br>pe_II_PDDEXK | RM_type_IIG;RM_Type_IIG<br>IV: RM_type_IV	
**GCF_001434365.1**	Limosilactobacillus gastricus DSM 16045	DSM 16045	Limosilactobacillus	0 / ND / 0	0 / ND / 0	0 / ND / 0	1 / C / 1	IV: RM_type_IV	
**GCF_001434705.1**	Lacticaseibacillus nasuensis JCM 17158	JCM 17158	Lacticaseibacillus	2 / C / 1	2 / C / 1	0 / ND / 0	1 / C / 1	I: RM_type_I;RM_Type_I<br>II: RM_type_II;D12_RM_Type_II_PDDEXK | RM_type_IIG;RM_Type_IIG<br>IV: RM_Type_IV	
**GCF_001435245.1**	Limosilactobacillus equigenerosi DSM 18793 = JCM 14505	DSM 18793	Limosilactobacillus	1 / C / 1	1 / C / 1	0 / ND / 0	1 / C / 1	I: RM_type_I;RM_Type_I<br>II: RM_Type_IIG<br>IV: RM_type_IV	
**GCF_001436135.1**	Lacticaseibacillus thailandensis DSM 22698 = JCM 13996	DSM 22698	Lacticaseibacillus	1 / C / 1	1 / C / 1	0 / ND / 0	1 / C / 1	I: RM_type_I;RM_Type_I<br>II: N6_N4_RM_Type_II_HATPase<br>IV: RM_Type_IV	
**GCF_001436905.1**	Pediococcus claussenii	DSM 14800	Pediococcus	0 / ND / 0	1 / C / 1	0 / ND / 0	1 / C / 1	II: RM_type_II<br>IV: RM_type_IV	
**GCF_001437055.1**	Limosilactobacillus secaliphilus	DSM 17896	Limosilactobacillus	0 / ND / 0	0 / ND / 0	0 / ND / 0	0 / ND / 0		
**GCF_001437425.1**	Weissella minor	DSM 20014	Weissella	0 / ND / 0	0 / ND / 0	0 / ND / 0	0 / ND / 0		
**GCF_001438705.1**	Weissella kandleri	DSM 20593	Weissella	0 / ND / 0	1 / C / 1	0 / ND / 0	0 / ND / 0	II: RM_type_II;RM_Type_II	
**GCF_001536305.1**	Leuconostoc gasicomitatum KG16-1	LEKG1	Leuconostoc	0 / ND / 0	1 / C / 1	0 / ND / 0	0 / ND / 0	II: RM_type_II	
**GCF_001618285.1**	Oenococcus oeni	DSPZS12	Oenococcus	0 / ND / 0	0 / ND / 0	0 / ND / 0	0 / ND / 0		
**GCF_001702215.1**	Pediococcus claussenii	TMW 2.53	Pediococcus	0 / ND / 0	1 / C / 1	0 / ND / 0	1 / C / 1	II: RM_type_II<br>IV: RM_type_IV	
**GCF_001702235.1**	Pediococcus claussenii	TMW 2.54	Pediococcus	0 / P / 0	1 / C / 1	0 / ND / 0	1 / C / 1	II: RM_type_II<br>IV: RM_type_IV	DFONLY:GCF_001702235.1:001|Type_I|PARTIAL_CAN<br>DIDATE|RM_Type_I
**GCF_001752505.1**	Oenococcus kitaharae	NRIC_0650	Oenococcus	0 / ND / 0	1 / C / 1	0 / ND / 0	0 / ND / 0	II: RM_type_IIG;RM_Type_IIG	
**GCF_001752515.1**	Oenococcus kitaharae	NRIC_0649	Oenococcus	0 / ND / 0	2 / C / 1	0 / ND / 0	0 / ND / 0	II: RM_type_II;RM_Type_II | RM_type_IIG;RM_Type_IIG	
**GCF_001752545.1**	Oenococcus kitaharae	NRIC_0647	Oenococcus	0 / ND / 0	1 / C / 1	0 / ND / 0	0 / ND / 0	II: RM_type_IIG;RM_Type_IIG	
**GCF_001761545.1**	Weissella soli	KACC 11848	Weissella	1 / C / 1	2 / C / 1	0 / ND / 0	0 / ND / 0	I: RM_type_I;N6_RM_Type_I<br>II: RM_type_II;RM_Type_II | RM_type_IIG;RM_Type_IIG	
**GCF_001767275.1**	Pediococcus acidilactici	ZPA017	Pediococcus	0 / ND / 0	1 / C / 1	1 / C / 1	1 / C / 1	II: RM_type_IIG;RM_Type_IIG<br>III: RM_type_III;RM_Type_III<br>IV: RM_type_IV	
**GCF_001888925.1**	Lactobacillus delbrueckii subsp. jakobsenii ZN7a-9 = DSM 26046	DSM 26046	Lactobacillus	0 / ND / 0	0 / ND / 0	0 / ND / 0	2 / C / 1	IV: RM_type_IV	

**GCF_001891125.1**	Leuconostoc suionicum	DSM 20241	Leuconostoc	1 / C / 1	1 / C / 1	1 / C / 1	0 / ND / 0	I: RM_type_I;N6_RM_Type_I<br>II: RM_type_II;RM_Type_II<br>III: RM_type_III;N6_N4_RM_Type_III	
**GCF_001932615.1**	Weissella jogaejeotgali	FOL01	Weissella	1 / C / 1	0 / U / NA	0 / ND / 0	1 / C / 1	I: N6_RM_Type_I<br>IV: RM_Type_IV	UNRESOLVED:GCF_001932615.1:3|UNRESOLVED|UNCERTAIN|RM_type_HNH
**GCF_001998805.1**	Leuconostoc garlicum	KFRI01	Leuconostoc	0 / ND / 0	0 / ND / 0	0 / ND / 0	1 / C / 1	IV: RM_Type_IV	
**GCF_002174215.1**	Pediococcus acidilactici	SRCM101189	Pediococcus	0 / ND / 0	0 / ND / 0	0 / ND / 0	1 / C / 1	IV: RM_type_IV	
**GCF_002950695.1**	Weissella koreensis	WiKim0080	Weissella	0 / ND / 0	0 / ND / 0	0 / ND / 0	0 / ND / 0		
**GCF_002970915.1**	Lactiplantibacillus koreensis	CBA3605	Lactiplantibacillus	0 / ND / 0	1 / C / 1	0 / ND / 0	0 / ND / 0	II: RM_Type_II	
**GCF_002970935.1**	Lactiplantibacillus kimchii	CBA3606	Lactiplantibacillus	0 / ND / 0	1 / C / 1	0 / ND / 0	1 / C / 1	II: RM_type_IIG<br>IV: RM_type_IV	
**GCF_003150935.1**	Lactobacillus apis	ESL0185	Lactobacillus	1 / C / 1	1 / C / 1	0 / ND / 0	1 / C / 1	I: RM_type_I;RM_Type_I<br>II: RM_Type_II<br>IV: RM_type_IV	
**GCF_003151025.1**	Lactobacillus kullabergensis	ESL0186	Lactobacillus	1 / C / 1	2 / C / 1	0 / ND / 0	1 / C / 1	I: RM_type_I;N6_RM_Type_I<br>II: RM_type_II;DNA_met_RM_Type_II_HATPase | RM_type_IIG;RM_Type_IIG<br>IV: RM_type_IV	
**GCF_003173695.1**	Lactobacillus helsingborgensis	ESL0183	Lactobacillus	0 / ND / 0	1 / C / 1	0 / ND / 0	1 / C / 1	II: RM_type_II;RM_Type_II<br>IV: RM_type_IV	
**GCF_003176835.1**	Lactococcus lactis subsp. lactis	14B4	Lactococcus	1 / C / 1	0 / ND / 0	0 / ND / 0	0 / ND / 0	I: RM_type_I;N6_RM_Type_I	
**GCF_003264795.1**	Oenococcus oeni	19	Oenococcus	0 / ND / 0	0 / ND / 0	0 / ND / 0	0 / ND / 0		
**GCF_003584685.1**	Lactobacillus paragasseri	JCM 5343	Lactobacillus	0 / ND / 0	0 / ND / 0	0 / ND / 0	0 / ND / 0		
**GCF_003641185.1**	Lactiplantibacillus pentosus	DSM 20314	Lactiplantibacillus	0 / ND / 0	1 / C / 1	0 / ND / 0	1 / C / 1	II: RM_type_IIG;RM_Type_IIG<br>IV: RM_Type_IV	
**GCF_003722275.1**	Lactococcus lactis	IL1403	Lactococcus	1 / C / 1	0 / ND / 0	0 / ND / 0	0 / ND / 0	I: RM_type_I;RM_Type_I	
**GCF_003999355.1**	Lactobacillus amylolyticus	L5	Lactobacillus	0 / ND / 0	0 / ND / 0	1 / C / 1	1 / C / 1	III: RM_Type_III<br>IV: RM_type_IV	
**GCF_004354645.1**	Leuconostoc fallax	ATCC 700006	Leuconostoc	0 / ND / 0	1 / C / 1	0 / ND / 0	0 / ND / 0	II: DNA_met_RM_Type_II_PLD	
**GCF_004377635.1**	Oenococcus oeni	a1	Oenococcus	1 / C / 1	1 / C / 1	0 / ND / 0	0 / ND / 0	I: RM_type_I;RM_Type_I<br>II: RM_type_II;RM_Type_II	
**GCF_005405405.1**	Lactiplantibacillus plajomi	NBRC 107333	Lactiplantibacillus	1 / C / 1	1 / C / 1	2 / C / 1	1 / C / 1	I: RM_type_I;RM_Type_I<br>II: D12_RM_Type_II_PDDEXK<br>III: RM_type_III;RM_Type_III<br>IV: RM_type_IV	
**GCF_005405465.1**	Weissella thailandensis	JCM 10695	Weissella	0 / ND / 0	1 / C / 1	0 / ND / 0	0 / ND / 0	II: RM_type_IIG;RM_Type_IIG_2	
**GCF_007954605.1**	Leuconostoc lactis	CBA3625	Leuconostoc	1 / C / 1	0 / ND / 0	0 / ND / 0	1 / C / 1	I: RM_type_I;N6_RM_Type_I<br>IV: RM_Type_IV	
**GCF_007954785.1**	Leuconostoc citreum	CBA3621	Leuconostoc	1 / C / 1	0 / ND / 0	0 / ND / 0	0 / ND / 0	I: RM_type_I;N6_RM_Type_I	
**GCF_008107645.1**	Leuconostoc litchii	MB7	Leuconostoc	0 / ND / 0	1 / C / 1	0 / ND / 0	0 / ND / 0	II: RM_Type_IIG	
**GCF_008831485.1**	Lactobacillus acetotolerans	LA749	Lactobacillus	0 / ND / 0	0 / ND / 0	0 / ND / 0	1 / C / 1	IV: RM_type_IV	
**GCF_009184845.1**	Lacticaseibacillus manihotivorans	LM010	Lacticaseibacillus	0 / P / 0	1 / C / 1	0 / ND / 0	1 / C / 1	II: RM_type_II;RM_Type_II<br>IV: RM_Type_IV	DFONLY:GCF_009184845.1:001|Type_I|PARTIAL_CANDIDATE|N6_RM_Type_I
**GCF_009856565.1**	Streptococcus thermophilus	EU01	Streptococcus	2 / C / 1	2 / C / 1	1 / C / 1	1 / C / 1	I: RM_type_I;RM_Type_I<br>II: DNA_met_RM_Type_II_PLD | RM_type_II;RM_Type_II<br>III: RM_type_III;RM_Type_III<br>IV: RM_type_IV	
**GCF_009913655.1**	Lactiplantibacillus plantarum	SRCM100442	Lactiplantibacillus	1 / C / 1	0 / ND / 0	0 / ND / 0	1 / C / 1	I: RM_type_I;RM_Type_I<br>IV: RM_Type_IV	
**GCF_009913995.1**	Pediococcus pentosaceus	SRCM102738	Pediococcus	0 / ND / 0	0 / ND / 0	0 / ND / 0	1 / C / 1	IV: RM_type_IV	
**GCF_009914015.1**	Pediococcus pentosaceus	SRCM102739	Pediococcus	0 / ND / 0	0 / ND / 0	0 / ND / 0	1 / C / 1	IV: RM_type_IV	
**GCF_009914035.1**	Pediococcus pentosaceus	SRCM102740	Pediococcus	0 / ND / 0	0 / ND / 0	0 / ND / 0	1 / C / 1	IV: RM_type_IV	
**GCF_010092385.1**	Pediococcus acidilactici	CACC 537	Pediococcus	1 / C / 1	0 / ND / 0	0 / ND / 0	1 / C / 1	I: RM_type_I;RM_Type_I<br>IV: RM_type_IV	
**GCF_011058775.1**	Lactobacillus iners	C0322A1	Lactobacillus	1 / C / 1	2 / C / 1	0 / ND / 0	0 / ND / 0	I: RM_type_I;N6_RM_Type_I<br>II: RM_type_II;DNA_met_RM_Type_II_PDDEXK | RM_type_IIG;RM_Type_IIG	DFONLY:GCF_011058775.1:002|Type_I|PARTIAL_CANDIDATE|N6_RM_Type_I
**GCF_011304355.1**	Weissella coleopterorum	HDW19	Weissella	0 / ND / 0	1 / C / 1	0 / ND / 0	0 / ND / 0	II: RM_type_IIG;RM_Type_IIG	
**GCF_013113695.1**	Oenococcus oeni	OE37	Oenococcus	0 / ND / 0	0 / ND / 0	0 / ND / 0	0 / ND / 0		
**GCF_013407595.1**	Lacticaseibacillus absianus	YH-lac23	Lacticaseibacillus	0 / ND / 0	2 / C / 1	0 / ND / 0	1 / C / 1	II: RM_type_II;RM_Type_II | RM_type_IIG;RM_Type_IIG<br>IV: RM_type_IV	
**GCF_014131735.1**	Lactiplantibacillus plantarum	DSM 20174	Lactiplantibacillus	0 / ND / 0	0 / ND / 0	0 / ND / 0	1 / C / 1	IV: RM_Type_IV	
**GCF_014145545.1**	Limosilactobacillus agrestis	BG-MG3-A	Limosilactobacillus	0 / P / 0	0 / ND / 0	0 / ND / 0	1 / C / 1	IV: RM_type_IV	SHARED:GCF_014145545.1:6|Type_I|PARTIAL_CANDIDATE|RM_type_I;N6_RM_Type_I
**GCF_014145555.1**	Limosilactobacillus albertensis	Lr3000	Limosilactobacillus	0 / ND / 0	5 / C / 1	0 / ND / 0	1 / C / 1	II: RM_type_II;RM_Type_II | RM_Type_IIG | RM_type_IIG;RM_Type_IIG<br>IV: RM_type_IV	
**GCF_014145615.1**	Limosilactobacillus balticus	BG-AF3-A	Limosilactobacillus	0 / ND / 0	0 / ND / 0	1 / C / 1	1 / C / 1	III: RM_type_III;RM_Type_III<br>IV: RM_type_IV	
**GCF_014397255.1**	Weissella diestrammenae	KACC 16890	Weissella	0 / ND / 0	0 / ND / 0	0 / ND / 0	0 / ND / 0		
**GCF_014656585.1**	Lactobacillus kefiranofaciens	1207	Lactobacillus	2 / C / 1	4 / C / 1	1 / C / 1	4 / C / 1	I: RM_type_I;N6_RM_Type_I | RM_type_I;RM_Type_I<br>II: RM_Type_IIG | RM_type_IIG;RM_Type_IIG | RM_type_IIG;RM_Type_IIG_2<br>III: RM_type_III;N6_N4_RM_Type_III<br>IV: GmrSD_RM_Type_IV | RM_type_IV	
**GCF_014789145.1**	Weissella viridescens	NJ100	Weissella	0 / ND / 0	1 / C / 1	0 / ND / 0	0 / ND / 0	II: RM_type_II;RM_Type_II	
**GCF_016127255.1**	Leuconostoc pseudomesenteroides	FDAARGOS_1003	Leuconostoc	1 / C / 1	1 / C / 1	0 / ND / 0	0 / ND / 0	I: RM_type_I;RM_Type_I<br>II: RM_type_II;RM_Type_II	
**GCF_016127815.1**	Pediococcus acidilactici	FDAARGOS_1007	Pediococcus	0 / ND / 0	0 / ND / 0	0 / ND / 0	1 / C / 1	IV: RM_type_IV	
**GCF_016647595.1**	Lactobacillus ultunensis	Kx293C1	Lactobacillus	2 / C / 1	0 / ND / 0	0 / ND / 0	2 / C / 1	I: RM_type_I;N6_RM_Type_I | RM_type_I;RM_Type_I<br>IV: RM_type_IV	
**GCF_016919665.1**	Lactococcus taiwanensis	K_LL001	Lactococcus	1 / C / 1	1 / C / 1	0 / ND / 0	0 / ND / 0	I: RM_type_I;N6_RM_Type_I<br>II: RM_type_IIG;RM_Type_IIG_2	
**GCF_017068355.1**	Lactococcus taiwanensis	K_LL004	Lactococcus	1 / C / 1	1 / C / 1	0 / ND / 0	0 / ND / 0	I: RM_type_I;RM_Type_I<br>II: RM_type_II	
**GCF_017894345.1**	Lactobacillus taiwanensis	CLG01	Lactobacillus	0 / ND / 0	2 / C / 1	0 / ND / 0	1 / C / 1	II: RM_type_II | RM_Type_IIG<br>IV: RM_type_IV	
**GCF_018394055.1**	Weissella ceti	CECT 7719	Weissella	0 / P / 0	1 / C / 1	0 / ND / 0	0 / ND / 0	II: RM_type_II;D12_RM_Type_II_PDDEXK	SHARED:GCF_018394055.1:4|Type_I|PARTIAL_CANDI

									DATE|RM_type_I;RM_Type_I
**GCF_018502505.1**	Weissella tructae	W-1	Weissella	0 / ND / 0	1 / C / 1	0 / ND / 0	0 / ND / 0	II: RM_type_II;RM_Type_II	
**GCF_019448175.1**	Pediococcus acidilactici	PMC202	Pediococcus	1 / C / 1	0 / ND / 0	0 / ND / 0	1 / C / 1	I: RM_type_I;RM_Type_I<br>IV: RM_type_IV	
**GCF_019469265.1**	Lactobacillus panisapium	ESL0416	Lactobacillus	0 / ND / 0	1 / C / 1	0 / ND / 0	1 / C / 1	II: RM_Type_IIG<br>IV: RM_type_IV	
**GCF_019576155.1**	Weissella confusa	LM1	Weissella	0 / ND / 0	2 / C / 1	1 / C / 1	0 / ND / 0	II: RM_type_II;D12_RM_Type_II_PDDEXK | RM_type_II;DNA_met_RM_Type_II_PDDEXK;N6_RM_<br>Type_II_PDDEXK<br>III: RM_type_III;N6_N4_RM_Type_III	
**GCF_019797825.1**	Limosilactobacillus panis	MT-01	Limosilactobacillus	0 / ND / 0	0 / ND / 0	1 / C / 1	1 / C / 1	III: RM_type_III;RM_Type_III<br>IV: RM_type_IV	
**GCF_020510245.1**	Oenococcus oeni	AWRIB429	Oenococcus	0 / ND / 0	0 / ND / 0	0 / ND / 0	0 / ND / 0		
**GCF_020784695.1**	Limosilactobacillus secundus	LR77	Limosilactobacillus	1 / C / 1	1 / C / 1	0 / ND / 0	1 / C / 1	I: RM_type_I;RM_Type_I<br>II: RM_Type_IIG<br>IV: RM_type_IV	
**GCF_020844005.1**	Streptococcus thermophilus	4078	Streptococcus	1 / C / 1	2 / C / 1	0 / ND / 0	0 / ND / 0	I: N6_RM_Type_I<br>II: DNA_met_RM_Type_II_PDDEXK | RM_type_II;DNA_met_RM_Type_II_PDDEXK	
**GCF_021378055.1**	Pediococcus pentosaceus	ST75/87BZ	Pediococcus	0 / ND / 0	0 / ND / 0	0 / ND / 0	2 / C / 1	IV: RM_type_IV	
**GCF_023169905.1**	Lactococcus cremoris	7-1	Lactococcus	0 / ND / 0	0 / ND / 0	0 / ND / 0	0 / ND / 0		
**GCF_023499275.1**	Lactococcus petauri	B1726	Lactococcus	0 / ND / 0	1 / C / 1	0 / ND / 0	0 / ND / 0	II: RM_type_II;RM_Type_II	
**GCF_023920145.1**	Oenococcus oeni	SD-2a	Oenococcus	1 / C / 1	4 / C / 1	0 / ND / 0	0 / ND / 0	I: RM_type_I;RM_Type_I<br>II: D12_RM_Type_II_PDDEXK | RM_Type_II | RM_type_II;RM_Type_II	
**GCF_024334065.1**	Streptococcus thermophilus	CH8	Streptococcus	2 / C / 1	0 / ND / 0	0 / ND / 0	0 / ND / 0	I: RM_type_I;RM_Type_I	
**GCF_024397795.1**	Lactobacillus intestinalis	DSM 6629	Lactobacillus	3 / C / 1	1 / C / 1	0 / ND / 0	1 / C / 1	I: RM_type_I;N6_RM_Type_I<br>II:<br>DNA_met_RM_Type_II_PDDEXK;N6_N4_RM_Type_II_<br>PDDEXK<br>IV: RM_type_IV	
**GCF_024718545.1**	Weissella fangxianensis	HBUAS51963	Weissella	0 / ND / 0	2 / C / 1	0 / ND / 0	0 / ND / 0	II: RM_type_IIG;RM_Type_IIG | RM_type_IIG;RM_Type_IIG_2	
**GCF_025311495.1**	Limosilactobacillus oris	AM_LB10	Limosilactobacillus	1 / C / 1	2 / C / 1	0 / ND / 0	1 / C / 1	I: RM_type_I;RM_Type_I<br>II: RM_type_II;RM_Type_II | RM_Type_IIG<br>IV: RM_type_IV	
**GCF_025490915.1**	Lacticaseibacillus parakribbianus	YH-lacS6	Lacticaseibacillus	1 / C / 1	0 / ND / 0	0 / ND / 0	0 / ND / 0	I: RM_type_I;RM_Type_I	
**GCF_025739325.1**	Streptococcus thermophilus	CNRZ1151	Streptococcus	0 / P / 0	1 / C / 1	0 / ND / 0	0 / ND / 0	II: RM_Type_IIG	DFONLY:GCF_025739325.1:001|Type_I|PARTIAL_CAN<br>DIDATE|N6_RM_Type_I
**GCF_025948295.1**	Streptococcus thermophilus	CNRZ385	Streptococcus	1 / C / 1	1 / C / 1	0 / ND / 0	1 / C / 1	I: RM_type_I;RM_Type_I<br>II: DNA_met_RM_Type_II_PLD<br>IV: RM_type_IV	
**GCF_026222675.1**	Lactiplantibacillus pentosus	OHF 23	Lactiplantibacillus	0 / ND / 0	1 / C / 1	0 / ND / 0	1 / C / 1	II: RM_type_IIG;RM_Type_IIG<br>IV: RM_Type_IV	
**GCF_028607025.1**	Limosilactobacillus portuensis	LV1276_C155	Limosilactobacillus	0 / ND / 0	1 / C / 1	0 / ND / 0	1 / C / 1	II: RM_type_IIG;RM_Type_IIG<br>IV: RM_type_IV	
**GCF_028621815.1**	Pediococcus acidilactici	SRCM102024	Pediococcus	0 / ND / 0	0 / ND / 0	0 / ND / 0	1 / C / 1	IV: RM_type_IV	
**GCF_028736235.1**	Lacticaseibacillus pabuli	KACC 23028	Lacticaseibacillus	0 / ND / 0	0 / ND / 0	0 / ND / 0	2 / C / 1	IV: RM_Type_IV	
**GCF_029025825.1**	Lactiplantibacillus paraplantarum	FL-8	Lactiplantibacillus	0 / ND / 0	0 / ND / 0	0 / ND / 0	1 / C / 1	IV: RM_Type_IV	
**GCF_029726355.1**	Lacticaseibacillus huelsenbergensis	DSM 115425	Lacticaseibacillus	1 / C / 1	0 / ND / 0	1 / C / 1	1 / C / 1	I: RM_type_I;RM_Type_I<br>III: RM_type_III;RM_Type_III<br>IV: RM_Type_IV	
**GCF_029854335.1**	Lactiplantibacillus plantarum	MY04	Lactiplantibacillus	0 / ND / 0	0 / ND / 0	0 / ND / 0	1 / C / 1	IV: RM_Type_IV	
**GCF_029866985.1**	Streptococcus thermophilus	DGCC7710	Streptococcus	1 / C / 1	0 / ND / 0	0 / ND / 0	1 / C / 1	I: RM_type_I;RM_Type_I<br>IV: RM_type_IV	DFONLY:GCF_029866985.1:001|Type_I|PARTIAL_CAN<br>DIDATE|N6_RM_Type_I
**GCF_030480545.1**	Lacticaseibacillus pantheris	CM2	Lacticaseibacillus	1 / C / 1	0 / ND / 0	0 / ND / 0	1 / C / 1	I: RM_type_I;RM_Type_I<br>IV: RM_Type_IV	
**GCF_030770265.1**	Lacticaseibacillus parahuelsenbergensis	NCIMB 15471	Lacticaseibacillus	1 / C / 1	1 / C / 1	0 / ND / 0	2 / C / 1	I: RM_type_I;RM_Type_I<br>II: RM_type_II<br>IV: GmrSD_RM_Type_IV | RM_Type_IV	
**GCF_030770295.1**	Lacticaseibacillus styriensis	NCIMB 15473	Lacticaseibacillus	0 / ND / 0	1 / C / 1	0 / ND / 0	1 / C / 1	II:<br>RM_type_II;D12_RM_Type_II_PDDEXK;N6_N4_RM_Ty<br>pe_II_PDDEXK<br>IV: RM_Type_IV	
**GCF_030770335.1**	Lacticaseibacillus zeae subsp. silagei	NCIMB 15474	Lacticaseibacillus	0 / ND / 0	0 / ND / 0	0 / ND / 0	1 / C / 1	IV: RM_Type_IV	
**GCF_032762665.1**	Oenococcus oeni	K19-3	Oenococcus	0 / P / 0	1 / C / 1	0 / ND / 0	0 / ND / 0	II: DNA_met_RM_Type_II_PDDEXK	DFONLY:GCF_032762665.1:001|Type_I|PARTIAL_CAN<br>DIDATE|N6_RM_Type_I
**GCF_033142085.1**	Oenococcus oeni	UCMA15129	Oenococcus	0 / P / 0	0 / ND / 0	0 / ND / 0	0 / ND / 0		DFONLY:GCF_033142085.1:001|Type_I|PARTIAL_CAN<br>DIDATE|RM_Type_I
**GCF_033570435.1**	Limosilactobacillus reuteri	P43	Limosilactobacillus	0 / ND / 0	0 / ND / 0	0 / ND / 0	1 / C / 1	IV: RM_type_IV	
**GCF_034703025.1**	Pediococcus acidilactici	AF2019	Pediococcus	0 / ND / 0	1 / C / 1	0 / ND / 0	1 / C / 1	II: RM_type_II;RM_Type_II<br>IV: RM_type_IV	
**GCF_035985965.1**	Lactococcus lactis subsp. lactis	C20	Lactococcus	0 / ND / 0	1 / C / 1	0 / ND / 0	0 / ND / 0	II: RM_Type_IIG_2	
**GCF_036246165.1**	Lactiplantibacillus paraxiangfangensis	142-6	Lactiplantibacillus	0 / ND / 0	1 / C / 1	0 / ND / 0	0 / ND / 0	II: RM_type_IIG;RM_Type_IIG	
**GCF_036246185.1**	Lactiplantibacillus paraxiangfangensis	231-9	Lactiplantibacillus	1 / C / 1	1 / C / 1	0 / ND / 0	0 / ND / 0	I: RM_type_I;RM_Type_I<br>II: RM_type_IIG;RM_Type_IIG	
**GCF_036246225.1**	Lacticaseibacillus salsurivasis	74-4	Lacticaseibacillus	0 / ND / 0	2 / C / 1	0 / ND / 0	0 / ND / 0	II: RM_type_II | RM_type_II;RM_Type_II	
**GCF_036327715.1**	Weissella paramesenteroides	MbWp-142	Weissella	0 / ND / 0	0 / ND / 0	0 / ND / 0	0 / ND / 0		
**GCF_037290055.1**	Lactiplantibacillus argentoratensis	D1	Lactiplantibacillus	1 / C / 1	0 / ND / 0	0 / ND / 0	0 / ND / 0	I: RM_type_I;N6_RM_Type_I	
**GCF_037891785.1**	Lactococcus petauri	R21-91A	Lactococcus	0 / ND / 0	1 / C / 1	0 / ND / 0	0 / ND / 0	II: RM_type_II;RM_Type_II	
**GCF_037892375.1**	Lactococcus formosensis	M04020401 EE5e5	Lactococcus	1 / C / 1	1 / C / 1	0 / ND / 0	0 / ND / 0	I: RM_type_I;N6_RM_Type_I<br>II: RM_type_II	
**GCF_038049945.1**	Streptococcus thermophilus	TCI125	Streptococcus	1 / C / 1	0 / ND / 0	0 / ND / 0	1 / C / 1	I: N6_RM_Type_I<br>IV: RM_type_IV	
**GCF_039645515.1**	Lactococcus petauri	R22-25 IC35	Lactococcus	1 / C / 1	0 / ND / 0	0 / ND / 0	0 / ND / 0	I: RM_type_I;N6_RM_Type_I	
**GCF_039645575.1**	Lactococcus formosensis	M20011502 FF20a2	Lactococcus	1 / C / 1	0 / ND / 0	0 / ND / 0	0 / ND / 0	I: RM_type_I;RM_Type_I	
**GCF_039783225.1**	Streptococcus thermophilus	CNRZ760	Streptococcus	2 / C / 1	1 / C / 1	0 / ND / 0	0 / ND / 0	I: RM_Type_I | RM_type_I;RM_Type_I<br>II: RM_Type_II	
**GCF_039783785.1**	Streptococcus thermophilus	CNRZ887	Streptococcus	1 / C / 1	1 / C / 1	0 / ND / 0	0 / ND / 0	I: N6_RM_Type_I<br>II: RM_Type_IIG	

**GCF_039955095.1**	Limosilactobacillus allomucosae	WILCCON 0051	Limosilactobacillus	1 / C / 1	2 / C / 1	1 / C / 1	2 / C / 1	I: RM_type_I;RM_Type_I<br>II: RM_type_IIG | RM_type_IIG;RM_Type_IIG<br>III: RM_type_III;RM_Type_III<br>IV: RM_type_IV	
**GCF_041225485.1**	Lactococcus ileimucosae	26-13	Lactococcus	1 / C / 1	1 / C / 1	1 / C / 1	0 / ND / 0	I: RM_type_I;N6_RM_Type_I<br>II: RM_type_II;RM_Type_II<br>III: RM_Type_III	
**GCF_041225605.1**	Lactococcus muris	20-218	Lactococcus	1 / C / 1	1 / C / 1	1 / C / 1	0 / ND / 0	I: RM_type_I;RM_Type_I<br>II: RM_type_IIG;RM_Type_IIG<br>III: RM_Type_III	
**GCF_041281755.1**	Weissella sagaensis	ZD-66	Weissella	0 / ND / 0	1 / C / 1	0 / ND / 0	0 / U / NA	II:<br>RM_type_II;D12_RM_Type_II_PDDEXK;N6_N4_RM_Ty<br>pe_II_PDDEXK	UNRESOLVED:GCF_041281755.1:5|UNRESOLVED|U<br>NCERTAIN|RM_type_HNH
**GCF_041509385.1**	Streptococcus thermophilus	S24750	Streptococcus	1 / C / 1	2 / C / 1	1 / C / 1	0 / ND / 0	I: N6_RM_Type_I<br>II: DNA_met_RM_Type_II_PDDEXK | RM_type_II;RM_Type_II<br>III: RM_type_III;N6_N4_RM_Type_III	
**GCF_041514285.1**	Streptococcus thermophilus	S24733	Streptococcus	1 / C / 1	2 / C / 1	1 / C / 1	0 / ND / 0	I: RM_Type_I<br>II: DNA_met_RM_Type_II_PDDEXK | RM_type_II;RM_Type_II<br>III: RM_type_III;N6_N4_RM_Type_III	
**GCF_041514295.1**	Streptococcus thermophilus	S24732	Streptococcus	1 / C / 1	2 / C / 1	1 / C / 1	0 / ND / 0	I: RM_Type_I<br>II: DNA_met_RM_Type_II_PDDEXK | RM_type_II;RM_Type_II<br>III: RM_type_III;RM_Type_III	
**GCF_041893975.1**	Streptococcus thermophilus	S24748	Streptococcus	1 / C / 1	2 / C / 1	1 / C / 1	0 / ND / 0	I: RM_Type_I<br>II: DNA_met_RM_Type_II_PDDEXK | RM_type_II;RM_Type_II<br>III: RM_type_III;RM_Type_III	
**GCF_042847315.1**	Lacticaseibacillus jixiensis	N163-3-2	Lacticaseibacillus	0 / ND / 0	0 / ND / 0	0 / ND / 0	1 / C / 1	IV: RM_Type_IV	
**GCF_042997415.1**	Lactobacillus mulieris	CM2267_MRS1	Lactobacillus	1 / C / 1	0 / ND / 0	0 / ND / 0	1 / C / 1	I: RM_type_I;N6_RM_Type_I<br>IV: RM_type_IV	SHARED:GCF_042997415.1:5|Type_I|PARTIAL_CANDI<br>DATE|RM_type_I;RM_Type_I
**GCF_045760565.1**	Lactococcus muris	GBG-M2	Lactococcus	2 / C / 1	1 / C / 1	0 / ND / 0	0 / ND / 0	I: RM_type_I;N6_RM_Type_I | RM_type_I;RM_Type_I<br>II: RM_type_IIG;RM_Type_IIG	
**GCF_045801155.1**	Leuconostoc falkenbergense	SAEED 24451	Leuconostoc	0 / ND / 0	2 / C / 1	0 / ND / 0	0 / ND / 0	II: RM_type_II;D12_RM_Type_II_PDDEXK | RM_type_II;RM_Type_II	
**GCF_045862825.1**	Lactiplantibacillus brownii	MH-1	Lactiplantibacillus	0 / ND / 0	1 / C / 1	0 / ND / 0	0 / ND / 0	II: RM_Type_IIG	
**GCF_046109915.1**	Lactobacillus helveticus	TCI357	Lactobacillus	0 / P / 0	0 / ND / 0	0 / ND / 0	2 / C / 1	IV: RM_type_IV	DFONLY:GCF_046109915.1:001|Type_I|PARTIAL_CAN<br>DIDATE|N6_RM_Type_I
**GCF_047344935.1**	Leuconostoc mesenteroides	F17	Leuconostoc	1 / C / 1	0 / ND / 0	1 / C / 1	1 / C / 1	I: N6_RM_Type_I<br>III: RM_Type_III<br>IV: RM_Type_IV	
**GCF_050407965.1**	Lactiplantibacillus pentosus	TBRC 20328	Lactiplantibacillus	1 / C / 1	0 / ND / 0	0 / ND / 0	0 / ND / 0	I: RM_type_I;RM_Type_I	
**GCF_050847345.1**	Lactiplantibacillus argentoratensis	12-27B	Lactiplantibacillus	0 / P / 0	0 / ND / 0	0 / ND / 0	0 / ND / 0		DFONLY:GCF_050847345.1:001|Type_I|PARTIAL_CAN<br>DIDATE|RM_Type_I
**GCF_051098045.1**	Lactiplantibacillus paraplantarum	IBB3436	Lactiplantibacillus	1 / C / 1	0 / ND / 0	0 / ND / 0	1 / C / 1	I: RM_type_I;N6_RM_Type_I<br>IV: RM_Type_IV	
**GCF_052569745.1**	Streptococcus thermophilus	UCCSt50	Streptococcus	1 / C / 1	2 / C / 1	0 / ND / 0	0 / ND / 0	I: RM_Type_I<br>II: RM_Type_II | RM_type_II;RM_Type_II	
**GCF_052570115.1**	Streptococcus thermophilus	F1B	Streptococcus	1 / C / 1	2 / C / 1	0 / ND / 0	0 / ND / 0	I: RM_Type_I<br>II: RM_Type_II | RM_type_II;DNA_met_RM_Type_II_PDDEXK	
**GCF_052571685.1**	Streptococcus thermophilus	F2A	Streptococcus	1 / C / 1	2 / C / 1	0 / ND / 0	0 / ND / 0	I: N6_RM_Type_I<br>II: RM_Type_II | RM_type_II;RM_Type_II	
**GCF_054073565.1**	Weissella cibaria	LB13201	Weissella	0 / ND / 0	2 / C / 1	0 / ND / 0	1 / C / 1	II: RM_type_II;D12_RM_Type_II_PDDEXK | RM_Type_IIG<br>IV: RM_type_IV	
**GCF_054331775.1**	Limosilactobacillus ingluviei	Ch9e	Limosilactobacillus	2 / C / 1	0 / ND / 0	0 / ND / 0	2 / C / 1	I: RM_type_I;N6_RM_Type_I<br>IV: RM_type_IV	
**GCF_054452875.2**	Lacticaseibacillus saniviri	TDH-8	Lacticaseibacillus	0 / ND / 0	2 / C / 1	0 / ND / 0	1 / C / 1	II: N6_N4_RM_Type_II_PDDEXK | RM_type_IIG;RM_Type_IIG<br>IV: RM_Type_IV	
**GCF_054784775.1**	Lacticaseibacillus sp. GG6-2	GG6-2	Lacticaseibacillus	0 / ND / 0	3 / C / 1	0 / ND / 0	2 / C / 1	II: RM_type_II;D12_RM_Type_II_PDDEXK | RM_type_II;RM_Type_II | RM_type_IIG<br>IV: RM_Type_IV	
**GCF_054785075.1**	Lacticaseibacillus suihuaensis	N149-9	Lacticaseibacillus	2 / C / 1	2 / C / 1	0 / ND / 0	0 / ND / 0	I: RM_type_I;N6_RM_Type_I | RM_type_I;RM_Type_I<br>II: RM_Type_IIG | RM_type_IIG;RM_Type_IIG	
**GCF_055180435.1**	Lactococcus cremoris	FAM 17886	Lactococcus	0 / ND / 0	1 / C / 1	0 / ND / 0	0 / ND / 0	II:<br>RM_type_II;DNA_met_RM_Type_II_PDDEXK;N6_RM_<br>Type_II_PDDEXK	
**GCF_055502585.1**	Lactobacillus kitasatonis	PLL-01	Lactobacillus	2 / C / 1	0 / ND / 0	0 / ND / 0	3 / C / 1	I: RM_type_I;N6_RM_Type_I | RM_type_I;RM_Type_I<br>IV: RM_type_IV	
**GCF_055755075.1**	Pediococcus pentosaceus	MIANGUAN2	Pediococcus	1 / C / 1	1 / C / 1	1 / C / 1	2 / C / 1	I: RM_type_I;RM_Type_I<br>II: RM_type_IIG;RM_Type_IIG<br>III: RM_type_III;RM_Type_III<br>IV: RM_type_IV	
**GCF_056269605.1**	Streptococcus thermophilus	ST250	Streptococcus	1 / C / 1	1 / C / 1	0 / ND / 0	1 / C / 1	I: RM_Type_I<br>II: RM_Type_II<br>IV: RM_type_IV	
**GCF_056676095.1**	Limosilactobacillus mucosae	CXY	Limosilactobacillus	0 / P / 0	1 / C / 1	0 / ND / 0	2 / C / 1	II: RM_Type_II<br>IV: RM_type_IV	DFONLY:GCF_056676095.1:001|Type_I|PARTIAL_CAN<br>DIDATE|N6_RM_Type_I
**GCF_057786735.1**	Streptococcus thermophilus	KKP4124	Streptococcus	2 / C / 1	0 / ND / 0	0 / ND / 0	0 / ND / 0	I: RM_type_I;RM_Type_I	
**GCF_058111805.1**	Leuconostoc suionicum	NIBL3969	Leuconostoc	0 / ND / 0	1 / C / 1	0 / ND / 0	0 / ND / 0	II: RM_type_II	
**GCF_059089415.1**	Pediococcus acidilactici	46A	Pediococcus	0 / ND / 0	0 / ND / 0	0 / ND / 0	1 / C / 1	IV: RM_type_IV	
**GCF_060305805.1**	Lactococcus lactis	LBMWF02	Lactococcus	0 / ND / 0	1 / C / 1	0 / ND / 0	0 / ND / 0	II: RM_type_II;RM_Type_II	
**GCF_060307215.1**	Lactococcus lactis	LBMWF01	Lactococcus	0 / ND / 0	1 / C / 1	0 / ND / 0	0 / ND / 0	II: RM_type_II;RM_Type_II	
**GCF_900016205.1**	Leuconostoc inhae	PL111	Leuconostoc	0 / ND / 0	1 / C / 1	0 / ND / 0	0 / ND / 0	II: RM_type_II;DNA_met_RM_Type_II_PLD	
**GCF_900094835.1**	Weissella bombi	R-53094	Weissella	0 / ND / 0	1 / C / 1	0 / ND / 0	0 / ND / 0	II: RM_type_IIG;RM_Type_IIG	
**GCF_900240275.1**	[Lactobacillus] timonensis	Marseille-P3825	Limosilactobacillus	1 / C / 1	1 / C / 1	0 / ND / 0	1 / C / 1	I: RM_type_I;N6_RM_Type_I<br>II: RM_type_IIG;RM_Type_IIG<br>IV: RM_type_IV;RM_Type_IV	SHARED:GCF_900240275.1:9|Type_I|PARTIAL_CANDI<br>DATE|RM_type_I;N6_RM_Type_I
**GCF_900519455.1**	Oenococcus oeni	UBOCC-A-315001	Oenococcus	0 / ND / 0	0 / ND / 0	1 / C / 1	0 / ND / 0	III: RM_type_III;RM_Type_III	
**GCF_900519475.1**	Oenococcus oeni	CRBO_1381	Oenococcus	0 / ND / 0	0 / ND / 0	0 / ND / 0	0 / ND / 0		
**GCF_900636965.1**	Lacticaseibacillus rhamnosus	NCTC13764	Lacticaseibacillus	1 / C / 1	1 / C / 1	0 / ND / 0	1 / C / 1	I: RM_type_I;RM_Type_I<br>II: RM_type_IIG;RM_Type_IIG<br>IV: RM_Type_IV	

<table>
<tr><th></th><th></th><th></th><th></th><th></th><th></th><th></th><th></th><th></th></tr>
<tr><td>GCF_903885985.1</td><td>Streptococcus thermophilus</td><td>STH_CIRM_18</td><td>Streptococcus</td><td>1 / C / 1</td><td>3 / C / 1</td><td>1 / C / 1</td><td>0 / ND / 0</td><td>I: N6_RM_Type_I
II: RM_Type_II | RM_type_II;RM_Type_II
III: RM_type_III;N6_N4_RM_Type_III</td></tr>
<tr><td>GCF_903886345.1</td><td>Streptococcus thermophilus</td><td>STH_CIRM_32</td><td>Streptococcus</td><td>1 / C / 1</td><td>3 / C / 1</td><td>1 / C / 1</td><td>0 / ND / 0</td><td>I: N6_RM_Type_I
II: DNA_met_RM_Type_II_PDDEXK |
RM_type_II;DNA_met_RM_Type_II_PDDEXK
III: RM_type_III;N6_N4_RM_Type_III</td></tr>
<tr><td>GCF_903886475.1</td><td>Streptococcus thermophilus</td><td>STH_CIRM_65</td><td>Streptococcus</td><td>1 / C / 1</td><td>1 / C / 1</td><td>1 / C / 1</td><td>0 / ND / 0</td><td>I: RM_type_I;RM_Type_I
II: RM_Type_IIG
III: RM_type_III;N6_N4_RM_Type_III</td></tr>
<tr><td>GCF_903891025.1</td><td>Streptococcus thermophilus</td><td>STH_CIRM_1055</td><td>Streptococcus</td><td>1 / C / 1</td><td>2 / C / 1</td><td>0 / ND / 0</td><td>0 / ND / 0</td><td>I: RM_Type_I
II: RM_Type_II | RM_type_II;RM_Type_II</td></tr>
<tr><td>GCF_903891205.1</td><td>Streptococcus thermophilus</td><td>STH_CIRM_1125</td><td>Streptococcus</td><td>1 / C / 1</td><td>0 / ND / 0</td><td>0 / ND / 0</td><td>1 / C / 1</td><td>I: RM_Type_I
IV: RM_type_IV</td></tr>
<tr><td>GCF_947539375.1</td><td>Lactobacillus isalae</td><td>AMBV1719</td><td>Lactobacillus</td><td>0 / ND / 0</td><td>0 / ND / 0</td><td>0 / ND / 0</td><td>1 / C / 1</td><td>IV: RM_type_IV</td></tr>
<tr><td>GCF_963243495.1</td><td>Lactiplantibacillus xiangfangensis</td><td>IMDO EBMM8</td><td>Lactiplantibacillus</td><td>0 / ND / 0</td><td>0 / ND / 0</td><td>0 / ND / 0</td><td>1 / C / 1</td><td>IV: RM_type_IV</td></tr>
<tr><td>GCF_963281025.1</td><td>Limosilactobacillus vaginalis</td><td>ena-SAMPLE-TAB-11-08-2023-
18:23:42:417-22881</td><td>Limosilactobacillus</td><td>0 / ND / 0</td><td>1 / C / 1</td><td>0 / ND / 0</td><td>1 / C / 1</td><td>II: RM_type_IIG;RM_Type_IIG
IV: RM_type_IV</td></tr>
<tr><td>GCF_964063315.1</td><td>Leuconostoc carnosum</td><td>Leuconostoc carnosum CIRM-
BIA2809</td><td>Leuconostoc</td><td>0 / ND / 0</td><td>0 / ND / 0</td><td>0 / ND / 0</td><td>0 / ND / 0</td><td></td></tr>
<tr><td>GCF_965136965.1</td><td>Lactiplantibacillus plajomi</td><td>CIP110952T</td><td>Lactiplantibacillus</td><td>1 / C / 1</td><td>1 / C / 1</td><td>1 / C / 1</td><td>0 / ND / 0</td><td>I: RM_type_I;RM_Type_I
II: D12_RM_Type_II_PDDEXK
III: RM_type_III;RM_Type_III</td></tr>
<tr><td>GCF_965286035.1</td><td>Leuconostoc pseudomesenteroides</td><td>HF-IBB-469
LPHFIBB469</td><td>Leuconostoc</td><td>2 / C / 1</td><td>1 / C / 1</td><td>0 / ND / 0</td><td>0 / ND / 0</td><td>I: RM_type_I;N6_RM_Type_I
II: RM_type_II;RM_Type_II</td></tr>
<tr><td>GCF_965286295.1</td><td>Lactiplantibacillus argentoratensis</td><td>UH016_15
LAUH01615</td><td>Lactiplantibacillus</td><td>1 / C / 1</td><td>1 / C / 1</td><td>0 / ND / 0</td><td>0 / ND / 0</td><td>I: RM_type_I;N6_RM_Type_I
II: RM_type_II;RM_Type_II</td></tr>
<tr><td>GCF_965286585.1</td><td>Limosilactobacillus fermentum</td><td>HF-IBB-205
LFHFIBB205</td><td>Limosilactobacillus</td><td>0 / ND / 0</td><td>3 / C / 1</td><td>0 / ND / 0</td><td>1 / C / 1</td><td>II: RM_Type_II | RM_type_II;N6_RM_Type_II_PDDEXK
| RM_Type_IIG
IV: RM_type_IV</td></tr>
<tr><td>GCF_982267005.1</td><td>Leuconostoc lactis</td><td>Leuconostoc_lactis_FME322</td><td>Leuconostoc</td><td>0 / ND / 0</td><td>0 / ND / 0</td><td>0 / ND / 0</td><td>1 / C / 1</td><td>IV: RM_Type_IV</td></tr>
</table>

Summary: accepted systems - Type I 101, Type II (including IIG) 162, Type III 33, Type IV 121. Positive genomes - Type I 86, Type II 112, Type III 30, Type IV 100. Two unresolved HNH candidates generate one Type II NA cell and one Type IV NA cell in the primary figure matrix.

S7. Exploratory phylogenetic clustering and sensitivity analyses

The phylogenetic distribution of predicted Type I–IV restriction–modification (R-M) systems was evaluated using a character-randomization framework [1], the accepted unrooted host phylogeny of 196 LAB genomes, and the independently curated binary type-presence matrix. The binary trait was 1 when an assembly contained at least one COMPLETE_PREDICTED locus of the corresponding type and 0 otherwise, including cells containing only partial candidates. One unresolved Type II cell and one unresolved Type IV cell were treated as unknown (NA), never as confirmed absence.

For each type, the minimum number of state changes on the fixed host-tree topology was computed by unit-cost binary Sankoff parsimony, allowing either state at unknown tips. Parsimony scores are root-independent and do not incorporate branch lengths. Two one-sided randomization tests evaluated whether the observed score was smaller than expected: (i) a global null permuting evaluable states among all tips while preserving the type-specific total prevalence and NA tip positions; and (ii) a within-genus null permuting states separately within each of the ten operational genera while preserving the observed genus-specific frequencies. Both models used 99,999 permutations per type, with a fixed base random seed of 1961008 and deterministic test-specific offsets. Monte Carlo p-values were calculated as (1 + the number of permuted scores less than or equal to the observed score)/(1 + 99,999). Benjamini–Hochberg false-discovery-rate correction was performed separately for the four R-M types within each null model.

Three sensitivity analyses assessed interpretive uncertainty. First, the two NA states were independently assigned either 0 or 1 and their type-specific tests repeated using 49,999 permutations per assignment and null model. Second, genome labels were exchanged within the eight exact-identical 100-marker alignment groups (22 genomes in total), in 1,000 random trials for each R-M type; this tests ambiguity among these tips but not uncertainty at deeper nodes. Third, to evaluate the effect of uneven strain sampling, 100 random species-balanced subsets were constructed by selecting one assembly for each of 110 operational species designations. Both global and within-genus tests were repeated on each subset with 1,999 permutations per type and null model, and the four p-values were independently FDR-corrected within each subset and null model. All analyses retained the original 196-genome tree as a fixed topology, treating omitted tips as unknown. Species-balanced subsampling necessarily reduces statistical power and changes dataset composition, so failure to reject in a subset does not establish the absence of phylogenetic structure.

The analysis tests topological clustering conditional on a purposively assembled panel and the inferred host tree; it is not a phylogenetic logistic regression. No specific gain/loss history, horizontal transfer, selection coefficient, or functional restriction phenotype can be inferred from these broad-type distributions. Although 1,000 IQ-TREE ultrafast bootstrap trees are included in the deposited dataset, tree-topology uncertainty was not propagated through these character-randomization tests. Calculations and sensitivity results are provided with the associated scripts and data.

[1] Maddison and Slatkin (1991), Null models for the number of evolutionary steps in a character on a phylogenetic tree, Evolution 45, 1184–1197, DOI: 10.1111/j.1558-5646.1991.tb04385.x.

Supplementary Table S2. Parsimony-based phylogenetic clustering of R–M Types I–IV under global and within-genus randomization null models (99,999 permutations per test).

R-M type	Null model	Evaluable genomes	Positive genomes	Minimum changes	Null mean	Null 95% interval	p	BH q
Type I	global	196	86	51	63.97789	57–71	0.00057	0.00076
Type I	within genus	196	86	51	51.52824	46–57	0.487	0.487
Type II	global	195	112	49	62.91786	56–70	9e-05	0.00018
Type II	within genus	195	112	49	62.18573	56–68	8e-05	0.00032
Type III	global	196	30	25	28.31424	26–30	0.02449	0.02449
Type III	within genus	196	30	25	27.9922	25–30	0.03704	0.049387
Type IV	global	195	100	26	65.27295	58–72	1e-05	4e-05
Type IV	within genus	195	100	26	31.83202	28–35	0.00254	0.00508

Supplementary Table S3. Phylogenetic-signal sensitivity to species-balanced sampling (100 independently selected 110-taxon subsets; 1,999 permutations per test).

R-M type	Global median p	Global median q	Global q<0.05 (of 100)	Within-genus median p	Within-genus median q	Within-genus q<0.05 (of 100)
Type I	0.2420	0.3277	0	0.4752	0.5663	0
Type II	0.1310	0.2390	2	0.0473	0.1850	6
Type III	0.5445	0.5445	0	0.5272	0.5547	0
Type IV	0.0005	0.0020	100	0.3245	0.5493	0
